# Supplementary material for: Nonequilibrium thermodynamics of the asymmetric Sherrington-Kirkpatrick model
Source: Nat Commun. 2023 Jun 23;14:3685. doi: 10.1038/s41467-023-39107-y (PMC10290083; doi:10.1038/s41467-023-39107-y)
Supplement: Supplementary file 1 — Supplementary Information [file 41467_2023_39107_MOESM1_ESM.pdf]

Nonequilibrium thermodynamics of the asymmetric Sherrington-Kirkpatrick model

Supplementary Information

Miguel Aguilera
BCAM – Basque Center for Applied Mathematics, Bilbao, Spain
IKERBASQUE, Basque Foundation for Science, Bilbao, Spain.
School of Engineering and Informatics, University of Sussex, Falmer, Brighton. United Kingdom

Masanao Igarashi
Graduate School of Engineering, Hokkaido University, Sapporo, Japan

Hideaki Shimazaki
Graduate School of Informatics, Kyoto University, Kyoto, Japan
Center for Human Nature, Artificial Intelligence, and Neuroscience (CHAIN), Hokkaido University, Sapporo, Japan

.

Supplementary Note 1: A path integral approach to the asymmetric SK model

In this Supplementary Note, we compute the generating functional of the asymmetric kinetic Ising model averaged over the quenched couplings with Gaussian distributions, known as the configurational average.

The probability density of a specific trajectory of the kinetic Ising model, $\mathbf{s}_{0:t} = \{\mathbf{s}_0, \mathbf{s}_1, \dots, \mathbf{s}_t\}$, is defined as

$$\begin{aligned} p(\mathbf{s}_{0:t}) &= \prod_{u=1}^{t} p(\mathbf{s}_u \mid \mathbf{s}_{u-1}) p(\mathbf{s}_0) \\ &= \exp\left[\beta \sum_{i,u} s_{i,u} h_{i,u} - \sum_{i,u} \log\left[2\cosh\left[\beta h_{i,u}\right]\right]\right] p(\mathbf{s}_0), \end{aligned} \tag{S1.1}$$

where

$$h_{i,u} = H_{i,u} + \sum_j J_{ij} s_{j,u-1}. \tag{S1.2}$$

Here the summation over u is taken for $u = 1, \dots, t$. For simplicity, we will assume that $p(\mathbf{s}_0)$ only contains one possible value, that is, the initial distribution is a Kronecker delta $p_0(\mathbf{s}) = \prod_i \delta\left[s_i, s_{i,0}\right]$. Although this enables us to ignore the term, the next steps are generalizable to any initial distributions.

The dynamics above straightforwardly describes a synchronous kinetic Ising model under time-dependent fields $H_{i,u}$ and asymmetric couplings J_{ij}. In Supplementary Note 2, we will show how this model and its solution can be generalized to cover both synchronous and asynchronous dynamics.

In equilibrium systems, the partition function provides the statistical moments of the equilibrium distribution. Here, to find the statistical properties expected from ensemble trajectories of the asymmetric SK model as well as its steady-state entropy production (Eq. 18), we introduce the following generating functional or dynamical partition function:

$$\begin{aligned} Z_t(\mathbf{g}) = &\sum_{\mathbf{s}_{0:t}} p(\mathbf{s}_{0:t}) \exp\Bigg[\sum_{i,u} g_{i,u} s_{i,u} \\ &+ \sum_{i,u} g_u^S \left(\beta s_{i,u} h_{i,u} - \log\left[2\cosh\left[\beta h_{i,u}\right]\right]\right) + \sum_{i,u} g_u^{S^r} \left(\beta s_{i,u-1} h_{i,u}^r - \log\left[2\cosh\left[\beta h_{i,u}^r\right]\right]\right)\Bigg] \\ = &\sum_{\mathbf{s}_{0:t}} \exp\Bigg[\sum_{i,u} s_{i,u}\beta h_{i,u} - \log\left[2\cosh\left[\beta h_{i,u}\right]\right] + \sum_{i,u} g_{i,u} s_{i,u} \\ &+ \sum_{i,u} g_u^S \left(s_{i,u}\beta h_{i,u} - \log\left[2\cosh\left[\beta h_{i,u}\right]\right]\right) + \sum_{i,u} g_u^{S^r} \left(s_{i,u-1}\beta h_{i,u}^r - \log\left[2\cosh\left[\beta h_{i,u}^r\right]\right]\right)\Bigg], \end{aligned} \tag{S1.3}$$

where $h^r_{i,u} = H_{i,u} + \sum_j J_{ij} s_{j,u} = h_{i,u+1} + H_{i,u} - H_{i,u+1}$. Note that $h_{i,u}$ have to be defined up to $t+1$ to recover the backwards trajectory. The terms $g_{i,u}$ are designed to obtain the moments and other statistics of the system, and g^S_u and $g^{S^r}_u$ are for its conditional and reversed conditional entropy terms at time u.

We will use this generating functional to calculate the statistical moments of various random variables. We will denote them using an average function described as:

$$\begin{aligned}\langle f(\cdot)\rangle_{\mathbf{g}} =& \sum_{\mathbf{s}_{0:t}} p(\mathbf{s}_{0:t}) f(\cdot) \exp\Bigg[\sum_{i,u} g_{i,u} s_{i,u} \\ &+ \sum_{i,u} g^S_u \left(\beta s_{i,u} h_{i,u} - \log\left[2\cosh\left[\beta h_{i,u}\right]\right]\right) + \sum_{i,u} g^{S^r}_u \left(\beta s_{i,u-1} h^r_{i,u} - \log\left[2\cosh\left[\beta h^r_{i,u}\right]\right]\right)\Bigg]. \end{aligned} \tag{S1.4}$$

For simplicity, we will denote $\langle f(\cdot)\rangle_{\mathbf{0}}$ simply as $\langle f(\cdot)\rangle$, recovering the statistical moments of the original system.

The configurational average over Gaussian couplings (Eq. 38) of the generating functional is computed as

$$[Z_t(\mathbf{g})]_{\mathbf{J}} = \int \prod_{i,j} dJ_{ij} p(J_{ij}) Z_t(\mathbf{g}). \tag{S1.5}$$

The configurational average can be solved using a path integral method. To obtain the path integral form, we first insert an appropriate delta integral for the effective fields of each unit for the time steps $u = 1, \ldots, t+1$ to the above equation:

$$\begin{aligned} 1 =& \int d\boldsymbol{\theta} \prod_{i,u} \delta\left[\theta_{i,u} - \beta h_{i,u}\right] \\ =& \frac{1}{(2\pi)^{N(t+1)}} \int d\boldsymbol{\theta} d\hat{\boldsymbol{\theta}} \exp\left[\sum_{i,u} \mathrm{i}\hat{\theta}_{i,u}(\theta_{i,u} - \beta H_{i,u} - \beta \sum_j J_{ij} s_{j.u-1})\right], \end{aligned} \tag{S1.6}$$

where $\boldsymbol{\theta}$ is the $N(t+1)$-dimensional vector composed of the effective fields $\theta_{i,u}$ ($i = 1, \ldots, N$ and $u = 1, \ldots, t+1$). $\hat{\boldsymbol{\theta}}$ is the $N(t+1)$-dimensional conjugate effective field, and we used a Dirac delta function $\delta\left[x - a\right] = \frac{1}{2\pi} \int_{-\infty}^{\infty} e^{\mathrm{i}\zeta(x-a)} d\zeta$. Note that, from now on, all summations and products involving the conjugate effective field $\hat{\boldsymbol{\theta}}$ (as well as the order parameters we introduce later) will be performed over the range $u = 1, \ldots, t+1$. Next, we replace $\beta h_{i,u}$ in Eq. S1.3 with the auxiliary variable $\theta_{i,u}$ as well as $\beta h^r_{i,u}$ of the reversed couplings at time u with an auxiliary variable $\vartheta_{i,u} = \theta_{i,u+1} + \beta(H_{i,u} - H_{i,u+1})$, and place them inside the integral with respect to $\boldsymbol{\theta}$ (i.e., we perform the operation, $f(a) = \int f(x)\delta\left[x - a\right] dx$). The configurational average is written as

$$\begin{aligned} [Z_t(\mathbf{g})]_{\mathbf{J}} =& \frac{1}{(2\pi)^{N(t+1)}} \int d\boldsymbol{\theta} d\hat{\boldsymbol{\theta}} \prod_{i,j} dJ_{ij} p(J_{ij}) \\ &\cdot \sum_{\mathbf{s}_{1:t}} \exp\Bigg[\sum_{i,u} s_{i,u}(g_{i,u} + \theta_{i,u}) - \log\left[2\cosh\theta_{i,u}\right] + \sum_{i,u} g^S_u (s_{i,u}\theta_{i,u} - \sum_{i,u} \log\left[2\cosh\theta_{i,u}\right]) \\ &+ \sum_{i,u} g^{S^r}_u (s_{i,u-1}\vartheta_{i,u} - \log\left[2\cosh\vartheta_{i,u}\right]) + \sum_{i,u} \mathrm{i}\hat{\theta}_{i,u}(\theta_{i,u} - \beta H_{i,u} - \beta \sum_j J_{ij} s_{j,u-1})\Bigg]. \end{aligned} \tag{S1.7}$$

Using the Gaussian integral formula $\int dx \frac{1}{\sqrt{2\pi b}}^r \exp\left[ax - \frac{(x-c)^2}{2b}\right] = \exp\left[ac + \frac{a^2}{2}b\right]$, the expectation of $\exp\left[aJ_{ij}\right]$ is computed as

$$\int dJ_{ij} p(J_{ij}) \exp\left[aJ_{ij}\right] = \exp\left[aJ_0/N + \frac{a^2}{2}\Delta J^2/N\right]. \tag{S1.8}$$

Hence the integral related to J_{ij} in Eq. S1.7 is computed as

$$\begin{aligned}
&\int \left[\prod_{i,j} dJ_{ij} p(J_{ij})\right] \exp\left[-\sum_{i,u} \mathrm{i}\hat{\theta}_{i,u}\beta \sum_j J_{ij} s_{j,u-1}\right] \\
=&\prod_{i,j}\left[\int dJ_{ij} p(J_{ij}) \exp\left[-\beta\left(\sum_u \mathrm{i}\hat{\theta}_{i,u} s_{j,u-1}\right) J_{ij}\right]\right. \\
=&\prod_{i,j}\exp\left[-\beta\left(\sum_u \mathrm{i}\hat{\theta}_{i,u} s_{j,u-1}\right)\frac{J_0}{N} + \beta^2\left(\sum_u \mathrm{i}\hat{\theta}_{i,u} s_{j,u-1}\right)^2 \frac{\Delta J^2}{2N}\right] \\
=&\prod_{i,j}\exp\left[-\frac{\beta J_0}{N}\sum_u \mathrm{i}\hat{\theta}_{i,u} s_{j,u-1} + \frac{\beta^2 \Delta J^2}{2N}\sum_{u,v} \mathrm{i}\hat{\theta}_{i,u} s_{j,u-1} \mathrm{i}\hat{\theta}_{i,v} s_{j,v-1}\right].
\end{aligned} \tag{S1.9}$$

Using this result, the Gaussian integral form of the partition function is given as

$$\begin{aligned}
[Z_t(\mathbf{g})]_{\mathbf{J}} =& \frac{1}{(2\pi)^{N(t+1)}} \int d\boldsymbol{\theta} d\hat{\boldsymbol{\theta}} \sum_{\mathbf{s}_{1:t}} \exp\left[\sum_{i,u} s_{i,u}(g_{i,u} + \theta_{i,u}) - \sum_{i,u} \log\left[2\cosh\theta_{i,u}\right]\right. \\
&+ \sum_{i,u} g_u^S \left(s_{i,u}\theta_{i,u} - \log\left[2\cosh\theta_{i,u}\right]\right) + \sum_{i,u} g_u^{S^r} \left(s_{i,u-1}\vartheta_{i,u} - \log\left[2\cosh\vartheta_{i,u}\right]\right) \\
&+ \sum_{i,u} \mathrm{i}\hat{\theta}_{i,u}(\theta_{i,u} - \beta H_{i,u}) - \sum_u N\beta J_0 \Big(\frac{1}{N}\sum_i \mathrm{i}\hat{\theta}_{i,u}\Big)\Big(\frac{1}{N}\sum_j s_{j,u-1}\Big) \\
&\left.+ \frac{\beta^2 \Delta J^2}{2N}\sum_{i,u}\left(\mathrm{i}\hat{\theta}_{i,u}\right)^2 + \sum_{u>v} N\beta^2\Delta J^2 \Big(\frac{1}{N}\sum_i \mathrm{i}\hat{\theta}_{i,u}\mathrm{i}\hat{\theta}_{i,v}\Big)\Big(\frac{1}{N}\sum_j s_{j,u-1}s_{j,v-1}\Big)\right].
\end{aligned} \tag{S1.10}$$

Note that, for the term of summation over u, v, we separated the $u = v$ terms from the rest, resulting in elimination of the spin variables because $s_{j,u-1}s_{j,u-1} = 1$.

S1.1. Gaussian integral and saddle node approximation

We will evaluate the aforementioned expression with a Gaussian integral and a saddle node approximation, and show that the saddle node solutions become order parameters. For this goal, we first give an outline of the derivation, and then apply the steps to the above equation.

Let C be a real value, and x and y be complex values. Eq. S1.10 contains the term in the form of $\exp[Cxy]$. We can represent this term by a double Gaussian integral (a pair of the Gaussian integral formulas) with the form:

$$\begin{aligned}
\exp[Cxy] =& \exp\left[\frac{C}{2}\left(\frac{1}{2}(x+y)^2 + \frac{1}{2}(\mathrm{i}(x-y))^2\right)\right] \\
=& \frac{C}{4\pi}\int dz_R dz_I \exp\left[\frac{C}{2}\left(-\frac{1}{2}z_R^2 - \frac{1}{2}z_I^2 + (x+y)z_R + \mathrm{i}(x-y)z_I\right)\right] \\
=& \frac{C}{4\pi}\int dz_R dz_I \exp\left[\frac{C}{2}\left(-\frac{1}{2}z_R^2 - \frac{1}{2}z_I^2 + x(z_R + \mathrm{i}z_I) + y(z_R - \mathrm{i}z_I)\right)\right].
\end{aligned} \tag{S1.11}$$

Because the integrand is an analytic function, we can change the contour of the path integral in the complex space so that it includes the saddle-point solution. This contour integration produces the original value, $\exp[Cxy]$. Therefore, z_R and z_I are no longer real values but can be complex values.

When x and y are random variables, we can approximate the expectation of $\exp[Cxy]$ by the saddle node solutions when the constant C is large:

$$\int p(x,y)\exp[Cxy]\,dxdy \approx \exp\left[\frac{C}{2}\left\{-\frac{1}{2}{z_R^*}^2 - \frac{1}{2}{z_I^*}^2 + \log\int p(x,y)\exp\left[x(z_R^* + \mathrm{i}z_I^*) + y(z_R^* - \mathrm{i}z_I^*)\right]dxdy\right\}\right], \tag{S1.12}$$

where z_R^* and z_I^* are the saddle-point solutions that extremize the contents of the braces {} in Eq. S1.12. These solutions are given by the following self-consistent equations:

$$z_R^* = \langle x + y \rangle \,, \tag{S1.13}$$

$$z_I^* = \mathrm{i} \langle x - y \rangle \,, \tag{S1.14}$$

where the bracket $\langle \cdot \rangle$ represents

$$\langle f(x,y) \rangle = \frac{\int p(x,y) \exp\left[x(z_R^* + \mathrm{i} z_I^*) + y(z_R^* - \mathrm{i} z_I^*)\right] f(x,y)\, dxdy}{\int p(x,y) \exp\left[x(z_R^* + \mathrm{i} z_I^*) + y(z_R^* - \mathrm{i} z_I^*)\right]\, dxdy} \tag{S1.15}$$

We reiterate that for the saddle-point solution z_I^* is derived from substituting the exponent by its Taylor expansion around the minimum, i.e., $f(z_I) = f(z_I^*) + \frac{1}{2} f''(z_I^*)(z_I - \mathrm{i}(x-y))^2 + \mathcal{O}((z_I - \mathrm{i}(x-y))^3)$, which in this case has an imaginary value.

To obtain a more intuitive saddle-point solution, we can perform a change of variables

$$z_1^* = \frac{1}{2}(z_R^* + \mathrm{i} z_I^*), \qquad z_2^* = \frac{1}{2}(z_R^* - \mathrm{i} z_I^*) \tag{S1.16}$$

$$z_R^* = z_1^* + z_2^*, \qquad z_I^* = \mathrm{i}(z_1^* - z_2^*), \tag{S1.17}$$

resulting in

$$\int p(x,y) \exp\left[Cxy\right] dxdy \approx \exp\left[\frac{C}{2}\left\{-z_1^* z_2^* + \log \int p(x,y) \exp\left[x z_1^* + y z_2^*\right] dxdy\right\}\right], \tag{S1.18}$$

and

$$z_1^* = \langle y \rangle \,, \tag{S1.19}$$

$$z_2^* = \langle x \rangle \tag{S1.20}$$

In summary, the process previously described consists of 1) introducing a pair of Gaussian integrals, 2) finding a saddle-point solution, and 3) performing a change of variable to recover a solution in terms of expectations of the original variables. We now repeat the process for the integral of the partition function.

(i) Gaussian integrals. First, we introduce Gaussian integrals by applying Eq. S1.11 to the quadratic terms in the partition function. Using $C = N\beta J_0$, $x_{u-1} = \frac{1}{N}\sum_j s_{j,u-1}$ and $y_u = -\frac{1}{N}\sum_i \mathrm{i}\hat{\theta}_{i,u}$, we obtain

$$\begin{aligned}
&\exp\left[\sum_u (-N\beta J_0)\Big(\frac{1}{N}\sum_i \mathrm{i}\hat{\theta}_{i,u}\Big)\Big(\frac{1}{N}\sum_j s_{j,u-1}\Big)\right] \\
&= \prod_u \exp\left[C x_{u-1} y_u\right] \\
&= \left(\frac{C}{4\pi}\right)^t \int \prod_u dM_u^+ dM_u^- \exp\left[\frac{C}{2}\left(-\frac{1}{2}(M_u^+)^2 - \frac{1}{2}(M_u^-)^2 + x_{u-1}(M_u^+ + \mathrm{i}M_u^-) + y_u(M_u^+ - \mathrm{i}M_u^-)\right)\right],
\end{aligned} \tag{S1.21}$$

where M_u^+ and M_u^- are real-valued integral variables. Similarly, using $C = \frac{1}{2}N\beta^2\Delta J^2$, $x_{u-1,v-1} = \frac{1}{N}\sum_j s_{j,u-1}s_{j,v-1}$, $y_{u,v} = \frac{1}{N}\sum_i \hat{\theta}_{i,u}\hat{\theta}_{i,v}$, we have

$$\begin{aligned}
&\exp\left[\sum_{u,v} N\frac{\beta^2\Delta J^2}{2}\Big(\frac{1}{N}\sum_i \mathrm{i}\hat{\theta}_{i,u}\mathrm{i}\hat{\theta}_{i,v}\Big)\Big(\frac{1}{N}\sum_j s_{j,u-1}s_{j,v-1}\Big)\right] \\
&= \prod_{u>v} \exp\left[C x_{u-1,v-1} y_{u,v}\right] \\
&= \left(\frac{C}{4\pi}\right)^{t(t-1)/2} \int \prod_{u>v} dQ_{u,v}^+ dQ_{u,v}^- \exp\left[\frac{C}{2}\left(-\frac{1}{2}(Q_{u,v}^+)^2 - \frac{1}{2}(Q_{u,v}^-)^2 + x_{u-1,v-1}(Q_{u,v}^+ + \mathrm{i}Q_{u,v}^-) + y_{u,v}(Q_{u,v}^+ - \mathrm{i}Q_{u,v}^-)\right)\right],
\end{aligned} \tag{S1.22}$$

where $Q_{u,v}^+$ and $Q_{u,v}^-$ are real values. Note that the products over u and v are performed over the range $1, \ldots, t+1$.

With these double Gaussian integrals and defining $d\mathbf{M} = \prod_u dM_u^+ dM_u^-$ and $d\mathbf{Q} = \prod_{u,v} dQ_{u,v}^+ dQ_{u,v}^-$ we can rewrite the partition function as

$$\begin{aligned}[Z_t(\mathbf{g})]_{\mathbf{J}} =& \frac{(N\beta J_0)^t (N\beta^2 \Delta J^2)^{t(t-1)/2}}{(4\pi)^{t(t+1)/2}} \int d\mathbf{M} d\mathbf{Q} \exp \Bigg[- N\beta J_0 \sum_u \frac{(M_u^+)^2 + (M_u^-)^2}{4} \\ & - N\beta^2 \Delta J^2 \sum_{u>v} \frac{(Q_{u,v}^+)^2 + (Q_{u,v}^-)^2}{4} + \log \sum_{\mathbf{s}_{1:t}} \int d\boldsymbol{\theta} d\hat{\boldsymbol{\theta}} \mathrm{e}^{\Phi(\mathbf{s}_{0:t}, \boldsymbol{\theta}, \mathbf{g}) + \Omega(\hat{\boldsymbol{\theta}}, \boldsymbol{\theta}, \mathbf{g})} \Bigg],\end{aligned} \tag{S1.23}$$

where the remaining terms related to the random variable $\mathbf{s}$ and $\boldsymbol{\theta}, \hat{\boldsymbol{\theta}}$ from the Gaussian integral can be separated into the terms

$$\begin{aligned}\Phi(\mathbf{s}_{0:t}, \boldsymbol{\theta}, \mathbf{g}) =& \sum_{i,u} (g_{i,u} + \theta_{i,u})\, s_{i,u} - \sum_{i,u} \log\left[2\cosh\left[\theta_{i,u}\right]\right] \\ & + \sum_{i,u} g_u^S \left(s_{i,u}\theta_{i,u} - \log\left[2\cosh\theta_{i,u}\right]\right) + \sum_{i,u} g_u^{S^r} \left(s_{i,u-1}\vartheta_{i,u} - \log\left[2\cosh\vartheta_{i,u}\right]\right) \\ & + \sum_{i,u} \beta J_0 \frac{M_u^+ + \mathrm{i} M_u^-}{2} s_{i,u-1} + \sum_{i,u>v} \beta^2 \Delta J^2 \frac{Q_{u,v}^+ + \mathrm{i} Q_{u,v}^-}{2} s_{i,u-1} s_{i,v-1},\end{aligned} \tag{S1.24}$$

$$\begin{aligned}\Omega(\hat{\boldsymbol{\theta}}, \boldsymbol{\theta}, \mathbf{g}) =& \sum_{i,u} (\theta_{i,u} - \beta H_{i,u} - \beta J_0 \frac{M_u^+ - \mathrm{i} M_u^-}{2}) \mathrm{i}\hat{\theta}_{i,u} \\ & + \frac{\beta^2 \Delta J^2}{2} \sum_{i,u} \left(\mathrm{i}\hat{\theta}_{i,u}\right)^2 + \beta^2 \Delta J^2 \sum_{i,u>v} \frac{Q_{u,v}^+ - \mathrm{i} Q_{u,v}^-}{2} \mathrm{i}\hat{\theta}_{i,u} \mathrm{i}\hat{\theta}_{i,v} - N(t+1)\log\left[2\pi\right].\end{aligned} \tag{S1.25}$$

Now, the next two steps for solving the integral is to find a saddle-point solution and perform a change of variables. In the next section, we find that the solutions result in the order parameters of the system.

(ii) saddle-point integral solution. The exponent of the integrand above is proportional to N, making it possible to evaluate the integral by steepest descent, giving the saddle-point solution as

$$\begin{aligned}[Z_t(\mathbf{g})]_{\mathbf{J}} =& \exp \Bigg[\Bigg\{ - N\beta J_0 \sum_u \frac{(M_u^+(\mathbf{g}))^2 + (M_u^-(\mathbf{g}))^2}{4} - N\beta^2 \Delta J^2 \sum_{u>v} \frac{(Q_{u,v}^+(\mathbf{g}))^2 + (Q_{u,v}^-(\mathbf{g}))^2}{4} \\ & + \log \sum_{\mathbf{s}_{1:t}} \int d\boldsymbol{\theta} d\hat{\boldsymbol{\theta}} \mathrm{e}^{\Phi(\mathbf{s}_{0:t}, \boldsymbol{\theta}, \mathbf{g}) + \Omega(\hat{\boldsymbol{\theta}}, \boldsymbol{\theta}, \mathbf{g})} \Bigg\} \Bigg],\end{aligned} \tag{S1.26}$$

where the optimal values $\mathbf{M}(\mathbf{g}), \mathbf{Q}(\mathbf{g})$ are chosen to extremize (maximize or minimize) the quantity between the braces $\{\}$. We introduced the dependence with $\mathbf{g}$ to denote the optimal values as the solution of $\mathbf{M}, \mathbf{Q}$ will be different for different values of $\mathbf{g}$. As in the solutions in Eqs. S1.13 and S1.14, the solutions $M_u^+(\mathbf{g}), Q_{u,v}^+(\mathbf{g})$ and $M_u^-(\mathbf{g}), Q_{u,v}^-(\mathbf{g})$ are combinations of the average statistics of the variables of interest (multiplied by the imaginary unit for the latter).

(iii) Change of the variables. Having order parameters in this form can be cumbersome. We simplify them to directly capture the average statistics of the system by performing a change of variables at the saddle-point solution as exemplified in Eq. S1.16, resulting in:

$$\mu_u(\mathbf{g}) = \frac{M_u^+(\mathbf{g}) + \mathrm{i} M_u^-(\mathbf{g})}{2}, \qquad m_{u-1}(\mathbf{g}) = \frac{M_u^+(\mathbf{g}) - \mathrm{i} M_u^-(\mathbf{g})}{2}, \tag{S1.27}$$

$$\rho_{u,v}(\mathbf{g}) = \frac{Q_{u,v}^+(\mathbf{g}) + \mathrm{i} Q_{u,v}^-(\mathbf{g})}{2}, \qquad q_{u-1,v-1}(\mathbf{g}) = \frac{Q_{u,v}^+(\mathbf{g}) - \mathrm{i} Q_{u,v}^-(\mathbf{g})}{2}, \tag{S1.28}$$

or equivalently

$$M_u^+(\mathbf{g}) = m_{u-1}(\mathbf{g}) + \mu_u(\mathbf{g}), \qquad M_u^-(\mathbf{g}) = \mathrm{i}(m_{u-1}(\mathbf{g}) - \mu_u(\mathbf{g})), \tag{S1.29}$$

$$Q_{u,v}^+(\mathbf{g}) = q_{u-1,v-1}(\mathbf{g}) + \rho_{u,v}(\mathbf{g}), \qquad Q_{u,v}^-(\mathbf{g}) = \mathrm{i}(q_{u-1,v-1}(\mathbf{g}) - \rho_{u,v}(\mathbf{g})), \tag{S1.30}$$

where now we expect all $\mathbf{m}(\mathbf{g}), \boldsymbol{\mu}(\mathbf{g}), \mathbf{q}(\mathbf{g}), \boldsymbol{\rho}(\mathbf{g})$ to be real-valued.

This results in

$$[Z_t(\mathbf{g})]_{\mathbf{J}} = \exp\left[\left\{ - N\beta J_0 \sum_u \mu_u(\mathbf{g}) m_{u-1}(\mathbf{g}) - N\beta^2 \Delta J^2 \sum_{u>v} \rho_{u,v}(\mathbf{g}) q_{u-1,v-1}(\mathbf{g}) + \log \sum_{\mathbf{s}_{1:t}} \int d\boldsymbol{\theta} d\hat{\boldsymbol{\theta}} \mathrm{e}^{\Phi(\mathbf{s}_{0:t},\boldsymbol{\theta},\mathbf{g})+\Omega(\hat{\boldsymbol{\theta}},\boldsymbol{\theta},\mathbf{g})} \right\}\right], \tag{S1.31}$$

where now $\mathbf{m}(\mathbf{g}), \boldsymbol{\mu}(\mathbf{g}), \mathbf{q}(\mathbf{g}), \boldsymbol{\rho}(\mathbf{g})$ are chosen to extremize the quantity between the braces. Also, we define the terms

$$\begin{aligned}\Phi(\mathbf{s}_{0:t}, \boldsymbol{\theta}, \mathbf{g}) =& \sum_{i,u} (g_{i,u} + \theta_{i,u})\, s_{i,u} - \sum_{i,u} \log\left[2\cosh\theta_{i,u}\right] + \sum_{i,u} g_u^S \left(s_{i,u}\theta_{i,u} - \log\left[2\cosh\theta_{i,u}\right]\right) \\ &+ \sum_{i,u} g_u^{S^r} \left(s_{i,u-1}\vartheta_{i,u} - \log\left[2\cosh\vartheta_{i,u}\right]\right) + \sum_{i,u} \beta J_0 \mu_u(\mathbf{g}) s_{i,u-1} + \sum_{i,u>v} \beta^2\Delta J^2 \rho_{u,v}(\mathbf{g}) s_{i,u-1} s_{i,v-1},\end{aligned} \tag{S1.32}$$

$$\begin{aligned}\Omega(\hat{\boldsymbol{\theta}}, \boldsymbol{\theta}, \mathbf{g}) =& \sum_{i,u} (\theta_{i,u} - \beta H_{i,u} - \beta J_0 m_{u-1}(\mathbf{g}))\mathrm{i}\hat{\theta}_{i,u} \\ &+ \frac{\beta^2\Delta J^2}{2} \sum_{i,u} \left(\mathrm{i}\hat{\theta}_{i,u}\right)^2 + \beta^2 \Delta J^2 \sum_{i,u>v} q_{u-1,v-1}(\mathbf{g}) \mathrm{i}\hat{\theta}_{i,u}\mathrm{i}\hat{\theta}_{i,v} - N(t+1)\log\left[2\pi\right].\end{aligned} \tag{S1.33}$$

Note that the summation of u or v related to the order parameters are performed over the range $1, \ldots, t+1$. Also note that integration over disordered connections has removed couplings between units and replaced them with same-unit temporal couplings $\boldsymbol{\rho}(\mathbf{g})$ and varying effective fields, which are also independent between units, resulting in a mean-field solution where the activity of different spins is independent.

In the next section, we specify the conditions of the extrema, from which we find that some of the extrema are the order parameters.

S1.2. Introduction of the order parameters

To obtain the values of the order parameters, we extremize the contents of the braces, finding

$$\frac{\partial \log\left[Z_t(\mathbf{g})\right]_{\mathbf{J}}}{\partial \mu_{u+1}(\mathbf{g})} = \beta J_0 \left(\sum_i \langle s_{i,u} \rangle_{*,\mathbf{g}} - N m_u(\mathbf{g}) \right) = 0; \qquad m_u(\mathbf{g}) = \frac{1}{N} \sum_i \langle s_{i,u} \rangle_{*,\mathbf{g}}, \tag{S1.34}$$

$$\frac{\partial \log\left[Z_t(\mathbf{g})\right]_{\mathbf{J}}}{\partial m_{u-1}(\mathbf{g})} = \beta J_0 \left(-\sum_i \left\langle \mathrm{i}\hat{\theta}_{i,u} \right\rangle_{*,\mathbf{g}} - N \mu_u(\mathbf{g}) \right) = 0; \qquad \mu_u(\mathbf{g}) = -\frac{1}{N} \sum_i \left\langle \mathrm{i}\hat{\theta}_{i,u} \right\rangle_{*,\mathbf{g}}, \tag{S1.35}$$

$$\frac{\partial \log\left[Z_t(\mathbf{g})\right]_{\mathbf{J}}}{\partial \rho_{u+1,v+1}(\mathbf{g})} = \beta^2\Delta J^2 \left(\sum_i \langle s_{i,u} s_{i,v} \rangle_{*,\mathbf{g}} - N q_{u,v}(\mathbf{g}) \right) = 0; \qquad q_{u,v}(\mathbf{g}) = \frac{1}{N} \sum_i \langle s_{i,u} s_{i,v} \rangle_{*,\mathbf{g}}, \tag{S1.36}$$

$$\frac{\partial \log\left[Z_t(\mathbf{g})\right]_{\mathbf{J}}}{\partial q_{u-1,v-1}(\mathbf{g})} = \beta^2\Delta J^2 \left(\sum_i \left\langle \mathrm{i}\hat{\theta}_{i,u} \mathrm{i}\hat{\theta}_{i,v} \right\rangle_{*,\mathbf{g}} - N \rho_{u,v}(\mathbf{g}) \right) = 0; \qquad \rho_{u,v}(\mathbf{g}) = \frac{1}{N} \sum_i \left\langle \mathrm{i}\hat{\theta}_{i,u} \mathrm{i}\hat{\theta}_{i,v} \right\rangle_{*,\mathbf{g}}, \tag{S1.37}$$

where we define

$$\begin{aligned}\langle f(\cdot) \rangle_{*,\mathbf{g}} =& \frac{\sum_{\mathbf{s}_{1:t}} \int d\boldsymbol{\theta} d\hat{\boldsymbol{\theta}} f(\cdot) \mathrm{e}^{\Phi(\mathbf{s}_{0:t},\boldsymbol{\theta},\mathbf{g})+\Omega(\hat{\boldsymbol{\theta}},\boldsymbol{\theta},\mathbf{g})}}{\sum_{\mathbf{s}_{1:t}} \int d\boldsymbol{\theta} d\hat{\boldsymbol{\theta}} \mathrm{e}^{\Phi(\mathbf{s}_{0:t},\boldsymbol{\theta},\mathbf{g})+\Omega(\hat{\boldsymbol{\theta}},\boldsymbol{\theta},\mathbf{g})}} \\ =& \left[Z_t(\mathbf{g})\right]_{\mathbf{J}}^{-1} \sum_{\mathbf{s}_{1:t}} \int d\boldsymbol{\theta} d\hat{\boldsymbol{\theta}} f(\cdot) \mathrm{e}^{\Phi(\mathbf{s}_{0:t},\boldsymbol{\theta},\mathbf{g})+\Omega(\hat{\boldsymbol{\theta}},\boldsymbol{\theta},\mathbf{g}) - N\beta J_0 \sum_u \mu_u(\mathbf{g}) m_{u-1}(\mathbf{g}) - N\beta^2\Delta J^2 \sum_{u>v} \rho_{u,v}(\mathbf{g}) q_{u-1,v-1}(\mathbf{g})}.\end{aligned} \tag{S1.38}$$

These equations are in concordance with Eqs. S1.19, S1.20. Here, $\mathbf{m}(\mathbf{g})$, $\boldsymbol{\mu}(\mathbf{g})$, $\mathbf{q}(\mathbf{g})$, and $\boldsymbol{\rho}(\mathbf{g})$ provide the saddle-point solution of the configurational average integral.

On the other hand, the configurational average of the generating functional holds relations similar to Eqs. 24, 25:

$$\frac{\partial\left[Z_t(\mathbf{g})\right]_{\mathbf{J}}}{\partial g_{i,u}} = \left[\langle s_{i,u}\rangle_{\mathbf{g}}\right]_{\mathbf{J}}, \tag{S1.39}$$

$$\frac{\partial^2\left[Z_t(\mathbf{g})\right]_{\mathbf{J}}}{\partial g_{i,u}\partial g_{j,v}} = \left[\langle s_{i,u}s_{j,v}\rangle_{\mathbf{g}}\right]_{\mathbf{J}}, \tag{S1.40}$$

where

$$\begin{aligned}\langle f(\cdot)\rangle_{\mathbf{g}} =& \sum_{\mathbf{s}_{0:t}} f(\cdot)p(\mathbf{s}_{0:t})\exp\Bigg[\sum_{i,u} g_{i,u}s_{i,u} \\ &+ \sum_{i,u} g_u^S\left(\beta s_{i,u}h_{i,u} - \log\left[2\cosh\left[\beta h_{i,u}\right]\right]\right) + \sum_{i,u} g_u^{S^r}\left(\beta s_{i,u-1}h^r_{i,u} - \log\left[2\cosh\left[\beta h^r_{i,u}\right]\right]\right)\Bigg].\end{aligned} \tag{S1.41}$$

In addition, we have the following identities that will be helpful in eliminating spurious solutions

$$\frac{\partial\left[Z_t(\mathbf{g})\right]_{\mathbf{J}}}{\partial H_{i,u}} = \beta\left(\left[\langle s_{i,u}\rangle_{\mathbf{g}}\right]_{\mathbf{J}} - \left[\langle\tanh\left[\beta h_{i,u}\right]\rangle_{\mathbf{g}}\right]_{\mathbf{J}}\right), \tag{S1.42}$$

$$\frac{\partial^2\left[Z_t(\mathbf{g})\right]_{\mathbf{J}}}{\partial H_{i,u}\partial H_{j,v}} = \beta\left(\frac{\partial\left[\langle s_{i,u}\rangle_{\mathbf{g}}\right]_{\mathbf{J}}}{\partial H_{j,v}} - \frac{\partial\left[\langle\tanh\left[\beta h_{i,u}\right]\rangle_{\mathbf{g}}\right]_{\mathbf{J}}}{\partial H_{j,v}}\right). \tag{S1.43}$$

Note that the equations above are equal to zero for $\mathbf{g}=\mathbf{0}$.

To derive the order parameters, we calculate the same partial derivatives using Eq. S1.31. The order parameter of the system given by Eq. S1.39 is calculated directly as

$$\begin{aligned}\frac{\partial\left[Z_t(\mathbf{g})\right]_{\mathbf{J}}}{\partial g_{i,u}} =& \Bigg(\langle s_{i,u}\rangle_{*,\mathbf{g}} + \beta J_0\sum_v \frac{\partial\mu_v(\mathbf{g})}{\partial g_{i,u}}\left(Nm_{v-1}(\mathbf{g}) - \sum_j\langle s_{j,v-1}\rangle_{*,\mathbf{g}}\right) \\ &+ \beta J_0\sum_v\frac{\partial m_{v-1}(\mathbf{g})}{\partial g_{i,u}}\left(N\mu_v(\mathbf{g}) - \sum_j\langle\theta_{j,v}\rangle_{*,\mathbf{g}}\right) \\ &+ \beta^2\Delta J^2\sum_v\frac{\partial\rho_{v,w}(\mathbf{g})}{\partial g_{i,u}}\left(Nq_{v-1,w-1}(\mathbf{g}) - \sum_j\langle s_{j,v-1}s_{j,w-1}\rangle_{*,\mathbf{g}}\right) \\ &+ \beta^2\Delta J^2\sum_v\frac{\partial q_{v-1,w-1}(\mathbf{g})}{\partial g_{i,u}}\left(N\rho_{v,w}(\mathbf{g}) - \sum_j\langle\theta_{j,v}\theta_{j,w}\rangle_{*,\mathbf{g}}\right)\Bigg)\left[Z_t(\mathbf{g})\right]_{\mathbf{J}} \\ =& \langle s_{i,u}\rangle_{*,\mathbf{g}}\left[Z_t(\mathbf{g})\right]_{\mathbf{J}}.\end{aligned} \tag{S1.44}$$

Similarly, we obtain

$$\frac{\partial\left[Z_t(\mathbf{g})\right]_{\mathbf{J}}}{\partial H_{i,u}} = -\beta\left\langle \mathrm{i}\hat{\theta}_{i,u}\right\rangle_{*,\mathbf{g}}\left[Z_t(\mathbf{g})\right]_{\mathbf{J}} \tag{S1.45}$$

$$\frac{\partial^2\left[Z_t(\mathbf{g})\right]_{\mathbf{J}}}{\partial g_{i,u}\partial g_{j,v}} = \langle s_{i,u}s_{j,v}\rangle_{*,\mathbf{g}}\left[Z_t(\mathbf{g})\right]_{\mathbf{J}} \tag{S1.46}$$

$$\frac{\partial^2\left[Z_t(\mathbf{g})\right]_{\mathbf{J}}}{\partial H_{i,u}\partial H_{j,v}} = \beta^2\left\langle \mathrm{i}\hat{\theta}_{i,u}\mathrm{i}\hat{\theta}_{j,v}\right\rangle_{*,\mathbf{g}}\left[Z_t(\mathbf{g})\right]_{\mathbf{J}} \tag{S1.47}$$

Here we should note that, as there is no coupling between units, for $i \neq j$ we have a factorized solution $[\langle s_{i,u}s_{j,v}\rangle] = \langle s_{i,u}s_{j,v}\rangle_{*,\mathbf{g}} = \langle s_{i,u}\rangle_{*,\mathbf{g}}\langle s_{j,v}\rangle_{*,\mathbf{g}}$.

Finally, by comparing the above derivatives with Eqs. S1.39, S1.40, S1.42, and S1.43 we obtain the order parameters:

$$m_u(\mathbf{g}) = [Z_t(\mathbf{g})]_{\mathbf{J}}^{-1} \sum_i \left[\langle s_{i,u}\rangle_{\mathbf{g}}\right]_{\mathbf{J}}, \tag{S1.48}$$

$$q_{u,v}(\mathbf{g}) = [Z_t(\mathbf{g})]_{\mathbf{J}}^{-1} \sum_i \left[\langle s_{i,u} s_{i,v}\rangle_{\mathbf{g}}\right]_{\mathbf{J}}, \tag{S1.49}$$

$$\mu_u(\mathbf{g}) = [Z_t(\mathbf{g})]_{\mathbf{J}}^{-1} \sum_i \left(\left[\langle s_{i,u}\rangle_{\mathbf{g}}\right]_{\mathbf{J}} - \left[\langle \tanh[\beta h_{i,u}]\rangle_{\mathbf{g}}\right]_{\mathbf{J}}\right), \tag{S1.50}$$

$$\rho_{u,v}(\mathbf{g}) = [Z_t(\mathbf{g})]_{\mathbf{J}}^{-1} \sum_i \beta^{-1} \left(\frac{\partial \left[\langle s_{i,u}\rangle_{\mathbf{g}}\right]_{\mathbf{J}}}{\partial H_{j,v}} - \frac{\partial \left[\langle \tanh[\beta h_{i,u}]\rangle_{\mathbf{g}}\right]_{\mathbf{J}}}{\partial H_{j,v}}\right). \tag{S1.51}$$

Note that, at $\mathbf{g} = \mathbf{0}$, $\mu_u(\mathbf{0}) = \rho_u(\mathbf{0}) = 0$. As well, notice that $[Z_t(\mathbf{0})]_{\mathbf{J}} = 1$, retrieving activation rates and delayed self-correlations as the order parameters of the system $m_u(\mathbf{0}), q_{u,v}(\mathbf{0})$. Below, we will drop the parenthesis for the order parameters at $\mathbf{g} = \mathbf{0}$, referring to these quantities as $m_u, q_{u,v}$.

Similarly, the forward and reverse entropy rates are calculated from the functions

$$\begin{aligned}\frac{\partial [Z_t(\mathbf{g})]_{\mathbf{J}}}{\partial g_u^S} &= \sum_i \langle s_{i,u}\theta_{i,u} - \log[2\cosh\theta_{i,u}]\rangle_{*,\mathbf{g}} [Z_t(\mathbf{g})]_{\mathbf{J}} \\ &= \sum_i \left[\langle (s_{i,u}\theta_{i,u} - \log[2\cosh\theta_{i,u}])\rangle_{\mathbf{g}}\right]_{\mathbf{J}},\end{aligned} \tag{S1.52}$$

$$\begin{aligned}\frac{\partial [Z_t(\mathbf{g})]_{\mathbf{J}}}{\partial g_u^{S^r}} &= \sum_i \langle (s_{i,u-1}\vartheta_{i,u} - \log[2\cosh\vartheta_{i,u}])\rangle_{*,\mathbf{g}} [Z_t(\mathbf{g})]_{\mathbf{J}} \\ &= \sum_i \left[\langle (s_{i,u-1}\vartheta_{i,u} - \log[2\cosh\vartheta_{i,u}])\rangle_{\mathbf{g}}\right]_{\mathbf{J}},\end{aligned} \tag{S1.53}$$

evaluated at $\mathbf{g} = \mathbf{0}$.

S1.3. Mean-field solutions

After solving the saddle-point integral, we have the following expression for computing relevant quantities in the system

$$[Z_t(\mathbf{g})]_{\mathbf{J}} = \sum_{\mathbf{s}_{1:t}} \int d\boldsymbol{\theta} d\hat{\boldsymbol{\theta}} \mathrm{e}^{\Phi(\mathbf{s}_{0:t},\boldsymbol{\theta},\mathbf{g}) + \Omega(\hat{\boldsymbol{\theta}},\boldsymbol{\theta},\mathbf{g})}. \tag{S1.54}$$

At this point, we want to remove the effective fields $\boldsymbol{\theta}$ and effective conjugate fields $\hat{\boldsymbol{\theta}}$. For this goal, (i) we first remove the effective conjugate fields $\hat{\boldsymbol{\theta}}$ by recovering the delta functions from their integral forms. Then, (ii) we revert the effective fields $\boldsymbol{\theta}$ by removing the delta function. This results in the mean-field (factorized) generating functional, from which we obtain the mean-field solutions of order parameters, conditional entropy, or entropy production.

(i) Removing effective conjugate fields We first remove the conjugate fields by recovering a delta function. We rewrite

$$\mathrm{e}^{\Omega(\hat{\boldsymbol{\theta}},\boldsymbol{\theta},\mathbf{g})} = \prod_i \frac{1}{(2\pi)^{N(t+1)}} \exp\left[\sum_u (\theta_{i,u} - \beta H_{i,u} - \beta J_0 m_{u-1}(\mathbf{g}))\mathrm{i}\hat{\theta}_{i,u} + \frac{\beta^2 \Delta J^2}{2} \sum_{u,v} q_{u-1,v-1}(\mathbf{g})\mathrm{i}\hat{\theta}_{i,u}\mathrm{i}\hat{\theta}_{i,v}\right], \tag{S1.55}$$

defining $q_{u-1,u-1} = 1$ and $q_{u-1,v-1} = q_{v-1,u-1}$ to obtain a symmetric matrix. Note that the saddle-node solution Eq. S1.36 was defined only for $u > v$.

We can remove the quadratic terms of $\boldsymbol{\theta}$ by applying $N(t+1)$-dimensional multivariate Gaussian integrals of the form

$$\mathrm{e}^{\frac{1}{2}\sum_{u,v} K_{u,v} x_u x_v} = \frac{1}{\sqrt{(2\pi)^t |K^{-1}|}} \int d\mathbf{z} \mathrm{e}^{-\frac{1}{2}\sum_{u,v} K_{u,v} z_u z_v + \sum_{u,v} K_{u,v} x_u z_v}, \tag{S1.56}$$

for $x_u = -\beta\Delta J \mathrm{i}\hat{\theta}_{i,u}$ and $K_{u,v} = q_{u-1,v-1}(\mathbf{g})$. Similarly, we can remove the quadratic terms of $\hat{\boldsymbol{\vartheta}}$ by applying N univariate Gaussian integrals, obtaining

$$
\begin{aligned}
\int d\hat{\boldsymbol{\theta}} \mathrm{e}^{\Omega(\hat{\boldsymbol{\theta}},\boldsymbol{\theta},\mathbf{0})} =& \frac{1}{(2\pi)^{N(t+1)}} \prod_i \int d\hat{\boldsymbol{\theta}} d\mathbf{z} p(\mathbf{z}) \\
& \cdot \exp\left[\sum_u \mathrm{i}\hat{\theta}_{i,u}(\theta_{i,u} - \beta H_{i,u} - \beta J_0 m_{u-1}) - \beta\Delta J \sum_{u,v} q_{u-1,v-1}(\mathbf{g})\mathrm{i}\hat{\theta}_{i,u} z_v \right] \\
=& \prod_i \int d\mathbf{z} p(\mathbf{z}) \prod_u \delta\left[\theta_{i,u} - \beta \overline{h}_{i,u}(\mathbf{z})\right],
\end{aligned} \tag{S1.57}
$$

where $\mathbf{z} = (z_1, \ldots, z_{t+1})$, and the distribution $p(\mathbf{z}) = \mathcal{N}(0, \boldsymbol{\Sigma})$ is a multivariate Gaussian with zero mean and inverse covariance $\boldsymbol{\Sigma}^{-1} = \mathbf{q}(\mathbf{g})$, and

$$
\overline{h}_{i,u}(\mathbf{z}) = H_{i,u} + J_0 m_{u-1}(\mathbf{g}) + \Delta J \sum_v z_v q_{u-1,v-1}(\mathbf{g}), \tag{S1.58}
$$

We can simplify the expressions above into

$$
\overline{h}_{i,u}(\xi_u) = H_{i,u} + J_0 m_{u-1}(\mathbf{g}) + \Delta J \xi_u, \tag{S1.59}
$$

with $\xi_u = \sum_v z_v q_{u-1,v-1}$. Let $\boldsymbol{\xi} = (\xi_1, \ldots, \xi_{t+1})$, then it follows $p(\boldsymbol{\xi}) = \mathcal{N}(0, \mathbf{q})$. Similarly, we can derive

$$
\overline{h}^r_{i,u}(\xi_{u+1}) = \overline{h}_{i,u}(\xi_u) + H_{i,u} - H_{i,u+1} = H_{i,u} + J_0 m_u + \Delta J \xi_{u+1}. \tag{S1.60}
$$

(ii) Removing effective fields We now revert the effective fields $\theta_{i,u}$ to $\beta\overline{h}_{i,u}(\mathbf{z})$ by removing the delta function, which replaces the original $\beta h_{i,u}$ with the mean-field equivalent.

Introducing the equivalences in the previous sections, we have

$$
\begin{aligned}
\mathrm{e}^{\Phi(\mathbf{s}_{0:t},\boldsymbol{\theta},\mathbf{g})} =& \prod_i \exp\left[\sum_u s_{i,u}(g_{i,u} + \theta_{i,u}) - \sum_u \log\left[2\cosh\theta_{i,u}\right] + \sum_u \beta s_{i,u-1}\widetilde{h}_{i,u-1} \right. \\
& \left. + \sum_u g^S_u \left(s_{i,u}\theta_{i,u} - \log\left[2\cosh\theta_{i,u}\right]\right) + \sum_u g^{S^r}_u \left(s_{i,u-1}\vartheta_{i,u} - \log\left[2\cosh\vartheta_{i,u}\right]\right) \right],
\end{aligned} \tag{S1.61}
$$

with

$$
\widetilde{h}_{i,u-1} = \sum_u J_0 \mu_u(\mathbf{g}) + \sum_{u>v} \beta\Delta J^2 \rho_{u,v}(\mathbf{g}) s_{i,v-1}. \tag{S1.62}
$$

Note that for $\mathbf{g} = \mathbf{0}$, $\widetilde{h}_{i,u-1}$ terms disappear.

This leads us to the mean-field solution of the configurational average of the generating functional

$$
\begin{aligned}
\left[Z_t(\mathbf{g})\right]_{\mathbf{J}} =& \int d\boldsymbol{\theta} \sum_{\mathbf{s}_{1:t}} \mathrm{e}^{\Phi(\mathbf{s}_{0:t},\boldsymbol{\theta},\mathbf{g})} \prod_i \int d\boldsymbol{\xi} p(\boldsymbol{\xi}) \prod_u \delta\left[\theta_{i,u} - \beta\overline{h}_{i,u}(\xi_u)\right] \\
=& \prod_i \sum_{\mathbf{s}_{i,1:t}} \int d\boldsymbol{\xi} p(\boldsymbol{\xi}) \exp\left[\sum_u s_{i,u}\left(g_{i,u} + \beta\overline{h}_{i,u}(\xi_u)\right) - \sum_u \log 2\cosh\left[\beta\overline{h}_{i,u}(\xi_u)\right] \right. \\
& + \sum_u \beta s_{i,u-1}\widetilde{h}_{i,u-1} + \sum_u \beta(g^S_u s_{i,u}\overline{h}_{i,u}(\xi_u) + g^{S^r}_u s_{i,u-1}\overline{h}^r_{i,u}(\xi_{u+1})) \\
& \left. - \sum_u \left(g^S_u \log\left[2\cosh\left[\beta\overline{h}_{i,t}(\xi_t)\right]\right] - g^{S^r}_u \log\left[2\cosh\left[\beta\overline{h}^r_{i,u}(\xi_{u+1})\right]\right] \right) \right].
\end{aligned} \tag{S1.63}
$$

The summation over u is taken for the range from 1 to t. With $\boldsymbol{\xi}$ defined in the range $u = 1, \ldots, t+1$, we can recover the values of $\overline{h}_{i,u}$ and $\overline{h}^r_{i,u}$ for all time steps.

Similarly, statistical moments are obtained as

$$\begin{aligned}\langle f(\cdot)\rangle_{*,\mathbf{0}} =&\prod_i \sum_{\mathbf{s}_{i,1:t}} \int d\boldsymbol{\xi} p(\boldsymbol{\xi}) f(\cdot) \exp \Bigg[\sum_u s_{i,u} \left(g_{i,u} + \beta \overline{h}_{i,u}(\xi_u) \right) - \sum_u \log 2\cosh \left[\beta \overline{h}_{i,u}(\xi_u) \right] \\ &+ \sum_u \beta s_{i,u-1} \widetilde{h}_{i,u-1} + \sum_u \beta (g_u^S s_{i,u} \overline{h}_{i,u}(\xi_u) + g_u^{S^r} s_{i,u-1} \overline{h}^r_{i,u}(\xi_{u+1})) \\ &- \sum_u \Big(g_u^S \log \left[2\cosh \left[\beta \overline{h}_{i,t}(\xi_t) \right] \right] - g_u^{S^r} \log \Big[2\cosh \Big[\beta \overline{h}^r_{i,u}(\xi_{u+1}) \Big] \Big] \Big) \Bigg]. \end{aligned} \tag{S1.64}$$

From this equation, we can derive the mean activation rate and the equal-time correlation of the ith unit. We note that the diagonal of the covariance matrix of $\boldsymbol{\xi}$ is equal to 1, hence we arrive at

$$[\langle s_{i,u} \rangle]_{\mathbf{J}} = \langle s_{i,u} \rangle_{*,\mathbf{0}} = \int \mathrm{D}z \tanh \left[\beta \overline{h}_{i,u}(z) \right], \tag{S1.65}$$

$$[\langle s_{i,u} s_{i,v} \rangle]_{\mathbf{J}} = \langle s_{i,u} s_{i,v} \rangle_{*,\mathbf{0}} = \int \mathrm{D}xy^{(q_{u-1,v-1})} \tanh \left[\beta \overline{h}_{i,u}(x) \right] \tanh \left[\beta \overline{h}_{i,v}(y) \right], \tag{S1.66}$$

where

$$\mathrm{D}z = \frac{1}{\sqrt{2\pi}} \mathrm{e}^{-\frac{1}{2} z^2}, \tag{S1.67}$$

$$\mathrm{D}xy^{(q)} = \frac{1}{2\pi \sqrt{1-q^2}} \mathrm{e}^{-\frac{x^2+y^2-2qxy}{2(1-q^2)}}. \tag{S1.68}$$

Finally, we obtain order parameters

$$m_u = \frac{1}{N} \sum_i [\langle s_{i,u} \rangle]_{\mathbf{J}} = \frac{1}{N} \sum_i \int \mathrm{D}z \tanh \left[\beta \left(H_{i,u} + J_0 m_{u-1} + \Delta J z \right) \right], \tag{S1.69}$$

$$q_{u,v} = \frac{1}{N} \sum_i [\langle s_{i,u} s_{i,v} \rangle]_{\mathbf{J}} = \frac{1}{N} \sum_i \int \mathrm{D}xy^{(q_{u-1,v-1})} \tanh \left[\beta \left(H_{i,u} + J_0 m_{u-1} + \Delta J x \right) \right] \tanh \left[\beta \left(H_{i,v} + J_0 m_{v-1} + \Delta J y \right) \right]. \tag{S1.70}$$

Note that magnetizations m_u are independent of $q_{u,v}$. This is consistent with findings of the asymmetric SK model lacking a spin-glass phase [25].

The conditional entropy results in

$$\begin{aligned} \left[S_{u|u-1} \right]_{\mathbf{J}} =& \sum_i \langle s_{i,u} \theta_{i,u} - \log \left[2\cosh \theta_{i,u} \right] \rangle_{*,\mathbf{0}} \\ =& \sum_i \int p(\boldsymbol{\xi}) \Big(\tanh \left[\beta \overline{h}_{i,u}(\xi_u) \right] \beta \overline{h}_{i,u}(\xi_u) - \log \left[2\cosh \left[\beta \overline{h}_{i,u}(\xi_u) \right] \right] \Big) \\ =& - \sum_i \int p(\boldsymbol{\xi}) \Big(\tanh \left[\beta \left(H_{i,u} + J_0 m_{u-1} + \Delta J \xi_u \right) \right] \beta \left(H_{i,u} + J_0 m_{u-1} + \Delta J \xi_u \right) \\ &- \log \left[2\cosh \left[\beta \left(H_{i,u} + J_0 m_{u-1} + \Delta J \xi_u \right) \right] \right] \Big) \\ =& - \sum_i \int \mathrm{D}z \Big(\beta \left(H_{i,u} + J_0 m_{u-1} \right) \tanh \left[\beta \left(H_{i,u} + J_0 m_{u-1} + \Delta J z \right) \right]) \\ &+ \beta^2 \Delta J^2 (1 - \tanh^2 \left[\beta \left(H_{i,u} + J_0 m_{u-1} + \Delta J z \right) \right]) \\ &- \log \left[2\cosh \left[\beta \left(H_{i,u} + J_0 m_{u-1} + \Delta J z \right) \right] \right] \Big). \end{aligned} \tag{S1.71}$$

Similarly, the reversed conditional entropy results in

$$
\begin{aligned}
\left[S^r_{u|u-1}\right]_{\mathbf{J}} =&\sum_i \langle (s_{i,u-1}\vartheta_{i,u} - \log\left[2\cosh\vartheta_{i,u}\right])\rangle_{*,\mathbf{0}} \\
=&\sum_i \int p(\boldsymbol{\xi})\Big(\tanh\left[\beta\overline{h}_{i,u-1}(\xi_u)\right] \beta\overline{h}^r_{i,u}(\xi_{u+1})) - \log\left[2\cosh\left[\beta\overline{h}^r_{i,u}(\xi_{u+1})\right]\right] \Big) \\
=&-\sum_i \int p(\boldsymbol{\xi})\Big(\tanh\left[\beta\left(H_{i,u-1} + J_0 m_{u-2} + \Delta J\xi_{u-1}\right)\right]\left(\beta\left(H_{i,u} + J_0 m_u + \Delta J\xi_{u+1}\right)\right)\Big) \\
&- \log\left[2\cosh\left[\beta\left(H_{i,u} + J_0 m_u + \Delta J\xi_{u+1}\right)\right]\right]\Big) \\
=&-\sum_i \int \mathrm{D}z\Big(\left(\beta H_{i,u} + \beta J_0 m_u\right)\tanh\left[\beta\left(H_{i,u-1} + J_0 m_{u-2} + \Delta J z\right)\right]) \\
&+ \beta^2\Delta J^2 q_{u,u-2}(1 - \tanh^2\left[\beta\left(H_{i,u-1} + J_0 m_{u-2} + \Delta J z\right)\right]) \\
&- \log\left[2\cosh\left[\beta\left(H_{i,u} + J_0 m_u + \Delta J z\right)\right]\right]\Big).
\end{aligned}
\tag{S1.72}
$$

where ξ_{u+1} is decomposed as a conditional Gaussian distribution for a given ξ_{u-1} as $\xi_{u+1} = q_{u,u-2}\xi_{u-1} + \sqrt{1 - q^2_{u,u-2}}\zeta_{u+1}$ where ζ_{u+1} is a normalized Gaussian independent of ξ_{u-1} and the term $q_{u,u-2}$ the covariance between variables.

Note that if the fields are constant $H_{i,u} = \Theta_i$, the system will converge to a steady state with $m_u = m$ and $q_{u,v} = q$ the entropy production simplifies to

$$
\left[\sigma_u\right]_{\mathbf{J}} = \beta^2\Delta J^2(1-q)\sum_i \int \mathrm{D}z(1 - \tanh^2\left[\beta\left(\Theta_i + J_0 m + \Delta J z\right)\right]),
\tag{S1.73}
$$

where m and q are the steady-state solutions of Eqs. S1.69 and S1.70 respectively. Note that, since every $q_{u,v}$ depends only on m_u and m_v, all $q_{u,v}$ converges to the same solution q under the steady state.

Supplementary Note 2: Solution of the asynchronous asymmetric SK models

The kinetic Ising system is sometimes written in the form of a continuous-time master equation with an asynchronous, stochastic update of spins [54]. Here we extend the discrete-time kinetic system with the synchronous updates in Supplementary Note 1 to the continuous-time model with asynchronous updates. We find the dynamical mean-field equation for the order parameters is effectively unchanged in the limit of long-term correlations, but short-term correlations decay slowly and the steady-state entropy production rate requires slight modification from the discrete counterpart.

First, we show that a system with (partially) asynchronous updates is equivalent to defining the time-dependent fields as a doubly stochastic process using independent Bernoulli random variables, $\tau_{i,u} \in \{0, 1\}$, which restrict updates of each spin i only to the times in which $\tau_{i,u} = 1$. Namely, we define a synchronous, discrete-time master equation

$$p(\mathbf{s}_u) = \sum_{\mathbf{s}_{u-1}} p(\mathbf{s}_u \,|\, \mathbf{s}_{u-1}) p(\mathbf{s}_{u-1}) \tag{S2.1}$$

driven by the following transition probabilities with time-dependent stochastic fields:

$$p(\mathbf{s}_u \,|\, \mathbf{s}_{u-1}) = \prod_i \frac{\exp\left[\beta s_{i,u} h_{i,u}\right]}{2\cosh\left[\beta h_{i,u}\right]}, \tag{S2.2}$$

$$h_{i,u} = H_{i,u} + \sum_j J_{ij} s_{j,u-1}, \tag{S2.3}$$

$$H_{i,u} = \Theta_{i,u} + (1 - \tau_{i,u}) K s_{i,u-1}. \tag{S2.4}$$

where $\tau_{i,u}$ are binary random variables independently following a Bernoulli distribution with rate α, i.e., $p(\tau_{i,u} = 1) = \alpha$. When $\tau_{i,u} = 1$, the spin updates normally with the field $\Theta_{i,u}$. When $\tau_{i,u} = 0$, the field is $K s_{i,u-1}$, i.e., coupled to the previous spin value with a strength K. If K is large, the current state is tightly coupled with the previous state. This means that the spin state is unchanged from the previous state in the limit $K \to \infty$. In this limit, the transition probability results in

$$p(s_{i,u} \,|\, \mathbf{s}_{u-1}) = \tau_{i,u} w(s_{i,u} \,|\, \mathbf{s}_{u-1}) + (1 - \tau_{i,u}) \delta\left[s_{i,u}, s_{i,u-1}\right]. \tag{S2.5}$$

where $w(s_{i,u} \,|\, \mathbf{s}_{u-1})$ is a transition rate given by

$$w(s_{i,u} \,|\, \mathbf{s}_{u-1}) = \frac{\exp\left[\beta s_{i,u} h^1_{i,u}\right]}{2\cosh\left[\beta h^1_{i,u}\right]}. \tag{S2.6}$$

$$h^1_{i,u} = \Theta_{i,u} + \sum_j J_{ij} s_{j,u-1}. \tag{S2.7}$$

We note that $\alpha = 1$ recovers the synchronous update in Supplementary Note 1. However, for a small α (such that $N\alpha \ll 1$), only one spin can update at a time and the system becomes equivalent to a single-spin update dynamics. Thus, we can model both the synchronous and asynchronous updates by these doubly-stochastic time-dependent fields.

Since only one spin can be updated at each time-step for a small probability α with the transfer rate $w(s_{i,u} \,|\, \mathbf{s}_{u-1})$, the master equation (equivalent to Eq. 7 in this condition) expected over $\tau_{i,u}$ is described as

$$\begin{aligned} p(\mathbf{s}_u) &= \sum_{\mathbf{s}_{u-1}} p(\mathbf{s}_u \,|\, \mathbf{s}_{u-1}) p(\mathbf{s}_{u-1}) \\ &= p_{u-1}(\mathbf{s}_u) + \sum_{\mathbf{s}_{u-1}} \left(p(\mathbf{s}_u \,|\, \mathbf{s}_{u-1}) p(\mathbf{s}_{u-1}) - p(\mathbf{s}_{u-1} \,|\, \mathbf{s}_u) p_{u-1}(\mathbf{s}_u) \right) \\ &= p_{u-1}(\mathbf{s}_u) + \alpha \sum_i \Big(w(s_{i,u} \,|\, \mathbf{s}^{[i]}_u) p_{u-1}(\mathbf{s}^{[i]}_u) - w(-s_{i,u} \,|\, \mathbf{s}_u) p_{u-1}(\mathbf{s}_u) \Big). \end{aligned} \tag{S2.8}$$

where the $^{[i]}$ operator flips the sign of the i-th spin (therefore, $\mathbf{s}_{u-1} = \mathbf{s}^{[i]}_u$), and the second term in the equation stands for the system's probability flow. We now represent the discrete time steps $u = 1, 2, \ldots$ on the real-valued line of the continuous time. We set the Bernoulli probability α ($\tau_{i,u}$ being 1) to be identical to the time resolution of the discrete-time step in the continuous time. The starting time of the u-th bin is given by $t = (u - 1)\alpha$. This condition

means that the time t is equivalent to the expected number of the spin updates up to the u-th bin. Note that t stands for the continuous time and should not be taken as the last discrete-time step that we use in the subscript of the variables. In the limit $\alpha \to 0$, the system is equivalent to a continuous time description for the new time variable t:

$$\frac{dp(\mathbf{s},t)}{dt} = \sum_i \Big(w(s_i \,|\, \mathbf{s}^{[i]}) p(\mathbf{s}^{[i]},t) - w(-s_i \,|\, \mathbf{s}) p(\mathbf{s},t) \Big) . \tag{S2.9}$$

This formulation leads to the definition of the entropy change as

$$\begin{aligned}\frac{d\sigma^{\mathrm{sys}}(t)}{dt} =& -\frac{d}{dt}\sum_{\mathbf{s}} p(\mathbf{s},t)\log p(\mathbf{s},t) \\ =& -\sum_{\mathbf{s}} \frac{dp(\mathbf{s},t)}{dt}\log p(\mathbf{s},t) - \sum_{\mathbf{s}} \frac{dp(\mathbf{s},t)}{dt} \\ =& -\sum_{\mathbf{s}} \frac{dp(\mathbf{s},t)}{dt}\log p(\mathbf{s},t)\end{aligned} \tag{S2.10}$$

Using Eq. S2.9, the entropy change is further written as

$$\begin{aligned}\frac{d\sigma^{\mathrm{sys}}(t)}{dt} =& -\sum_{\mathbf{s}}\sum_i \Big(w(s_i \,|\, \mathbf{s}^{[i]}) p(\mathbf{s}^{[i]},t) - w(-s_i \,|\, \mathbf{s}) p(\mathbf{s},t) \Big) \log p(\mathbf{s},t) \\ =& -\frac{1}{2}\sum_{\mathbf{s}}\sum_i \Big(w(s_i \,|\, \mathbf{s}^{[i]}) p(\mathbf{s}^{[i]},t) - w(-s_i \,|\, \mathbf{s}) p(\mathbf{s},t) \Big) \log p(\mathbf{s},t) \\ &+\frac{1}{2}\sum_{\mathbf{s}}\sum_i \Big(w(-s_i \,|\, \mathbf{s}) p(\mathbf{s},t) - w(s_i \,|\, \mathbf{s}^{[i]}) p(\mathbf{s}^{[i]},t) \Big) \log p(\mathbf{s},t) \\ =& -\frac{1}{2}\sum_{\mathbf{s}}\sum_i \Big(w(s_i \,|\, \mathbf{s}^{[i]}) p(\mathbf{s}^{[i]},t) - w(-s_i \,|\, \mathbf{s}) p(\mathbf{s},t) \Big) \log p(\mathbf{s},t) \\ &+\frac{1}{2}\sum_{i}\sum_{\mathbf{s}^{[i]}} \Big(w(s_i \,|\, \mathbf{s}^{[i]}) p(\mathbf{s}^{[i]},t) - w(-s_i \,|\, \mathbf{s}) p(\mathbf{s},t) \Big) \log p(\mathbf{s}^{[i]},t) \\ =& \frac{1}{2}\sum_{\mathbf{s}}\sum_i \Big(w(s_i \,|\, \mathbf{s}^{[i]}) p(\mathbf{s}^{[i]},t) - w(-s_i \,|\, \mathbf{s}) p(\mathbf{s},t) \Big) \log \frac{p(\mathbf{s}^{[i]},t)}{p(\mathbf{s},t)} .\end{aligned} \tag{S2.11}$$

To obtain the third equality, we applied the change of variables from $\mathbf{s}$ to $\mathbf{s}^{[i]}$ in the second term, which makes s_i into $-s_i$. We then reverted the summation over i and $\mathbf{s}^{[i]}$ to $\mathbf{s}$ and i since both sum all the states and flipping to obtain the fourth equality. From this form, the entropy change can be decomposed into an entropy production rate and entropy flow rate terms as follows [39]:

$$\begin{aligned}\frac{d\sigma^{\mathrm{sys}}(t)}{dt} =& \underbrace{\frac{1}{2}\sum_{\mathbf{s}}\sum_i \Big(w(s_i \,|\, \mathbf{s}^{[i]}) p(\mathbf{s}^{[i]},t) - w(-s_i \,|\, \mathbf{s}) p(\mathbf{s},t) \Big) \log \frac{w(s_i \,|\, \mathbf{s}^{[i]}) p(\mathbf{s}^{[i]},t)}{w(-s_i \,|\, \mathbf{s}) p(\mathbf{s},t)}}_{\text{Entropy production rate}} \\ &+ \underbrace{\frac{1}{2}\sum_{\mathbf{s}}\sum_i \Big(w(s_i \,|\, \mathbf{s}^{[i]}) p(\mathbf{s}^{[i]},t) - w(-s_i \,|\, \mathbf{s}) p(\mathbf{s},t) \Big) \log \frac{w(-s_i \,|\, \mathbf{s})}{w(s_i \,|\, \mathbf{s}^{[i]})}}_{\text{Entropy flow}} .\end{aligned} \tag{S2.12}$$

Thus, we define the continuous-time steady-state entropy production rate as:

$$\begin{aligned}\frac{d\sigma(t)}{dt} =& \frac{1}{2}\sum_{\mathbf{s}}\sum_i \Big(w(s_i \,|\, \mathbf{s}^{[i]}) p(\mathbf{s}^{[i]},t) - w(-s_i \,|\, \mathbf{s}) p(\mathbf{s},t) \Big) \log \frac{w(s_i \,|\, \mathbf{s}^{[i]}) p(\mathbf{s}^{[i]},t)}{w(-s_i \,|\, \mathbf{s}) p(\mathbf{s},t)} \\ =& \sum_{\mathbf{s}}\sum_i w(s_i \,|\, \mathbf{s}^{[i]}) p(\mathbf{s}^{[i]},t) \log \frac{w(s_i \,|\, \mathbf{s}^{[i]}) p(\mathbf{s}^{[i]},t)}{w(-s_i \,|\, \mathbf{s}) p(\mathbf{s},t)} .\end{aligned} \tag{S2.13}$$

S2.1. Generating functional

Knowing that we obtain the continuous-time asynchronous updates in the limit of $\alpha \to 0$ and $K \to \infty$, in what follows, we will derive the order parameters and entropy production rate with the asynchronous updates in continuous-time by first augmenting the generating functional defined in the discrete-time steps using the doubly stochastic fields and then applying these limits.

Given the augmented fields (Eq. S2.4), the effective field (Eq. S1.59) in the thermodynamic limit is decomposed as

$$\overline{h}_{i,u}(\xi_u) = \tau_{i,u}\overline{h}^{(1)}_{i,u}(\xi_u) + (1-\tau_{i,u})\overline{h}^{(0)}_{i,u}(\xi_t), \tag{S2.14}$$

where

$$\overline{h}^{(1)}_{i,u}(\xi_u) = \Theta_{i,u} + J_0 m_{u-1} + \Delta J \xi_u, \tag{S2.15}$$

$$\overline{h}^{(0)}_{i,u}(\xi_u) = K\left(s_{i,u-1} + \frac{1}{K}\left(\Theta_{i,u} + J_0 m_{u-1} + \Delta J \xi_u\right)\right). \tag{S2.16}$$

Given that K is large, we can approximate $h^{(0)}_{i,u}(\xi_u) \approx K s_{i,u-1}$ because the remaining two terms become negligible for calculating the spin update. Thus we have

$$\overline{h}_{i,u}(\xi_u) \approx \tau_{i,u}\overline{h}^{(1)}_{i,u}(\xi_u) + (1-\tau_{i,u})K s_{i,u-1}. \tag{S2.17}$$

for large K.

Using the aforementioned $\overline{h}_{i,u}(\xi_u)$, the system averaged over variables $\tau_{i,u}$ is now described by the following configurational average of a generating functional:

$$\begin{aligned}
[Z_t(\mathbf{g})]_{\mathbf{J},\boldsymbol{\tau}} =& \prod_i \sum_{\mathbf{s}_{i,1:t}} \sum_{\boldsymbol{\tau}_{i,1:t}} \int d\boldsymbol{\xi} p(\boldsymbol{\tau}_{i,1:t}) \exp\Bigg[\sum_u s_{i,u}\left(g_{i,u} + \beta\overline{h}_{i,u}(\xi_u)\right) - \sum_u \log 2\cosh\left[\beta\overline{h}_{i,u}(\xi_u)\right] \\
&+ \sum_u \beta(g^S_u s_{i,u}\overline{h}_{i,u}(\xi_u) + g^{S^r}_u s_{i,u-1}\overline{h}^r_{i,u}(\xi_{u+1})) \\
&- \sum_u \left(g^S_u \log\left[2\cosh\left[\beta\overline{h}_{i,t}(\xi_t)\right]\right] - g^{S^r}_u \log\left[2\cosh\left[\beta\overline{h}^r_{i,u}(\xi_{u+1})\right]\right]\right)\Bigg].
\end{aligned} \tag{S2.18}$$

The order parameters will be obtained similarly as in the general solution with additional averaging over the Bernoulli random variables. To simplify further steps, we will consider the configurational average of the mean and delayed correlation of N individual spins:

$$m_{i,u} = [\langle s_{i,u}\rangle]_{\mathbf{J},\boldsymbol{\tau}} = \lim_{\mathbf{g}\to\mathbf{0}} \frac{\partial[Z_t(\mathbf{g})]_{\mathbf{J},\boldsymbol{\tau}}}{\partial g_{i,u}} = \sum_{\boldsymbol{\tau}_{i,1:t}} p(\boldsymbol{\tau}_{i,1:t}) \int \mathrm{D}z \tanh\left[\beta\overline{h}_{i,u}(z)\right], \tag{S2.19}$$

$$q_{i,u,v} = [\langle s_{i,u}s_{i,v}\rangle]_{\mathbf{J},\boldsymbol{\tau}} = \lim_{\mathbf{g}\to\mathbf{0}} \frac{\partial^2[Z_t(\mathbf{g})]_{\mathbf{J},\boldsymbol{\tau}}}{\partial g_{i,u}\partial g_{i,v}} = \sum_{\boldsymbol{\tau}_{i,1:t}} p(\boldsymbol{\tau}_{i,1:t}) \int \mathrm{D}xy^{(q_{u-1,v-1})} \tanh\left[\beta\overline{h}_{i,u}(x)\right] \tanh\left[\beta\overline{h}_{i,v}(y)\right], \tag{S2.20}$$

which constitute the order parameters

$$m_u = \frac{1}{N}\sum_i m_{i,u}, \tag{S2.21}$$

$$q_{u,v} = \frac{1}{N}\sum_i q_{i,u,v}. \tag{S2.22}$$

More specifically, since the order parameters m_u and $q_{u,v}$ are given by the expectations over the independent random

variables $\tau_{i,u} \sim \text{Bernoulli}(\alpha)$, we further separate $m_{i,u}, q_{i,u,v}$ into the cases where $\tau_{i,u}, \tau_{i,v}$ are updated or not:

$$\begin{aligned} m_{i,u} =& \sum_{\tau_{i,u}} p(\tau_{i,u}) m_{i,u}^{\tau_{i,u}} \\ =& \sum_{\tau_{i,u}} p(\tau_{i,u}) \int \mathrm{D}z \tanh\left[\beta \overline{h}_{i,u}^{(\tau_{i,u})}(\xi_u)\right], \end{aligned} \tag{S2.23}$$

$$\begin{aligned} q_{i,u,v} =& \sum_{\tau_{i,u},\tau_{i,v}} p(\tau_{i,u}) p(\tau_{i,v}) q_{i,u,v}^{\tau_{i,u},\tau_{i,v}} \\ =& \sum_{\tau_{i,u},\tau_{i,v}} p(\tau_{i,u}) p(\tau_{i,v}) \int \mathrm{D}xy^{(q_{u-1,v-1})} \tanh\left[\beta \overline{h}_{i,u}^{(\tau_{i,u})}(x)\right] \tanh\left[\beta \overline{h}_{i,v}^{(\tau_{i,v})}(y)\right], \end{aligned} \tag{S2.24}$$

where $m_{i,u}^{0} = m_{i,u-1}$ and $q_{i,u,v}^{0,0} = q_{i,u-1,v-1}$.

Mean activation rate order parameter

First, we look into the mean activation rate. Since $p(\tau_{i,u} = 1) = \alpha$, the expectation of this order parameter by τ_i is given by

$$m_u = \alpha \frac{1}{N} \sum_i \int \mathrm{D}z \tanh\left[\beta\left(\Theta_{i,u} + J_0 m_{u-1} + \Delta J z\right)\right] + (1-\alpha) m_{u-1}, \tag{S2.25}$$

where we assumed $K \to \infty$ to obtain the second term. The above equation gives the dynamical mean-field equation in the discrete time steps. We now represent the discrete time steps $u = 1, 2, \ldots$ on the real-valued line of the continuous time using $t = (u-1)\alpha$. By representing the mean activation rate at the $u-1$th bin by $m(t)$, i.e., $m(t) = m_{u-1}$, the equation above is described as:

$$\frac{m(t+\alpha) - m(t)}{\alpha} = \frac{1}{N} \sum_i \int \mathrm{D}z \tanh\left[\beta\left(\Theta_i(t(+J_0 m(t) + \Delta J z)\right] - m(t) \tag{S2.26}$$

Hence, we obtain the following differential equation in the limit of $\alpha \to 0$:

$$\frac{dm(t)}{dt} = -m(t) + \frac{1}{N} \sum_i \int \mathrm{D}z \tanh\left[\beta\left(\Theta_i(t) + J_0 m(t) + \Delta J z\right)\right]. \tag{S2.27}$$

Note that Eqs. S2.25 converges to the same formula given by Eq. S1.69 when the magnetic fields are equal to $\Theta_{i,u}$.

Delayed self-correlation order parameter

Next, we compute the order parameter of the delayed self-correlation. We can simplify the decomposition of $q_{i,u,v}$ in two steps as:

$$q_{i,u,v} = \sum_{\tau_{i,v}} q_{i,u,v}^{\tau_{i,v}} p(\tau_{i,v}), \tag{S2.28}$$

$$q_{i,u,v}^{\tau_{i,v}} = \sum_{\tau_{i,u}} q_{i,u,v}^{\tau_{i,u},\tau_{i,v}} p(\tau_{i,u}), \tag{S2.29}$$

where the variable $q_{i,u,v}^{\tau_{i,v}}$ captures the marginal order parameter when we know $\tau_{i,v}$, i.e., if the spin at time v has been updated or not, regardless of whether the spin at time u has been updated or not. $q_{i,u,v}^{\tau_{i,u},\tau_{i,v}}$ is the order parameter given that we know both the update occurred at u and v.

We can decompose $q_{i,u,v}$ into the two cases, where spin v is or is not updated:

$$\begin{aligned} q_{i,u,v} =& (1-\alpha) q_{i,u,v}^{0} + \alpha q_{i,u,v}^{1}, \\ =& (1-\alpha) q_{i,u,v-1} + \alpha q_{i,u,v}^{1}. \end{aligned} \tag{S2.30}$$

Here we used the equivalence $q^0_{i,u,v} = q_{i,u,v-1}$ under $K \to \infty$ because, if the spin at time v is not updated, it takes the value of the spin at the previous time $v-1$, at which we do not know if the spin is updated or not. We then calculate the other variable $q^1_{i,u,v}$ in terms of the updates of u and v:

$$\begin{aligned} q^1_{i,u,v} =&(1-\alpha) q^{0,1}_{i,u,v} + \alpha q^{1,1}_{i,u,v} \\ =&(1-\alpha) q^1_{i,u-1,v} + \alpha \int \mathrm{D}xy^{(q_{u-1,v-1})} \tanh\left[\beta\left(\Theta_{i,u} + J_0 m_{u-1} + \Delta J x\right)\right] \tanh\left[\beta\left(\Theta_{i,v} + J_0 m_{v-1} + \Delta J y\right)\right]. \end{aligned} \tag{S2.31}$$

Here we used $q^{0,1}_{i,u,v} = q^1_{i,u-1,v}$ for the same reason previously mentioned, applied at time u. $q^{1,1}_{i,u,v}$ is the self-delayed correlation $q_{i,u,v}$ as in Eq. S2.20 with $H_{i,u} = \Theta_{i,u}$ and $H_{i,v} = \Theta_{i,v}$ because the spin was updated at these time steps with correlation between effective fields $q_{u-1,v-1}$, and there are no constraints from the previous spins.

Similarly to the mean activation rate, in the small $\alpha \to 0$ limit, with $t' \equiv \alpha(u-1)$, $t \equiv \alpha(v-1)$, the previous equations lead to

$$\frac{dq_i(t',t)}{dt} = - q_i(t',t) + q^1_i(t',t), \tag{S2.32}$$

$$\frac{dq^1_i(t',t)}{dt'} = - q^1_i(t',t) + \int \mathrm{D}xy^{(q(t',t))} \tanh\left[\beta\left(\Theta_i(t') + J_0 m(t') + \Delta J x\right)\right] \tanh\left[\beta\left(\Theta_i(t) + J_0 m(t) + \Delta J y\right)\right], \tag{S2.33}$$

The equations above are solved iteratively with boundary conditions:

$$\begin{aligned} q_i(t,t) =& q^1_i(t,t), = 1, \\ q_i(t',t) =& q_i(t,t'), \\ q^1_i(t',t) =& q^1_i(t,t'), \\ t' \geq 0&, t \geq 0. \end{aligned} \tag{S2.34}$$

Similar boundary conditions apply for the discrete time description in u, v. The delayed self-correlation order parameter $q(t',t)$ is obtained as the average of $q_i(t',t)$ over the spins. Similarly to the mean activation rate, the delayed self-correlation converges to Eq. S1.70 obtained under the synchronous update.

Long-range limit

Assuming a time-independent $\Theta_{i,u} (= \Theta_i)$, we can calculate the convergence values of $q_{i,u,v}$ in two steps, separating the dynamics in u and the dynamics in v. First, from Eq. S2.31, it is easy to see that, knowing $q_{u-1,v-1}$ for all values of u and a fixed v, the convergence point for $u \gg v$ will be

$$\begin{aligned} q^1_{i,\infty,v} \equiv& \lim_{u\to\infty} q^1_{i,u,v} \\ =& \lim_{u\to\infty} \int \mathrm{D}xy^{(q_{u-1,v-1})} \tanh\left[\beta\left(\Theta_i + J_0 m_{u-1} + \Delta J x\right)\right] \tanh\left[\beta\left(\Theta_i + J_0 m_{v-1} + \Delta J y\right)\right], \end{aligned} \tag{S2.35}$$

or, in continuous time

$$q^1_i(\infty,t) \equiv \lim_{t'\to\infty} \int \mathrm{D}xy^{(q_{t',t})} \tanh\left[\beta\left(\Theta_i + J_0 m(t') + \Delta J x\right)\right] \tanh\left[\beta\left(\Theta_i + J_0 m(t) + \Delta J y\right)\right]. \tag{S2.36}$$

Now we can solve the dynamics in v independently to u, writing Eq. S2.30 for $u \gg v$ as

$$q_{i,\infty,v} = (1-\alpha) q_{i,\infty,v-1} + \alpha q^1_{i,\infty,v}, \tag{S2.37}$$

as well as its continuous-time equivalent

$$\frac{dq_i(\infty,t)}{dt} = - q_i(\infty,t) + q^1_i(\infty,t). \tag{S2.38}$$

If we assume a starting point $q_{i,\infty,v}$ for a given v, then we find that $q_{i,\infty,v}$ converges for a large v (still under $u \gg v$) to

$$\begin{aligned} q_{i,\infty,\infty} \equiv& \lim_{v\to\infty} q_{i,\infty,v} = \lim_{v\to\infty} q^1_{i,\infty,v} \\ =& \lim_{v\to\infty} \lim_{u\to\infty} \int \mathrm{D}xy^{(q_{i,u-1,v-1})} \tanh\left[\beta\left(\Theta_i + J_0 m_{u-1} + \Delta J x\right)\right] \tanh\left[\beta\left(\Theta_i + J_0 m_{v-1} + \Delta J y\right)\right]. \end{aligned} \tag{S2.39}$$

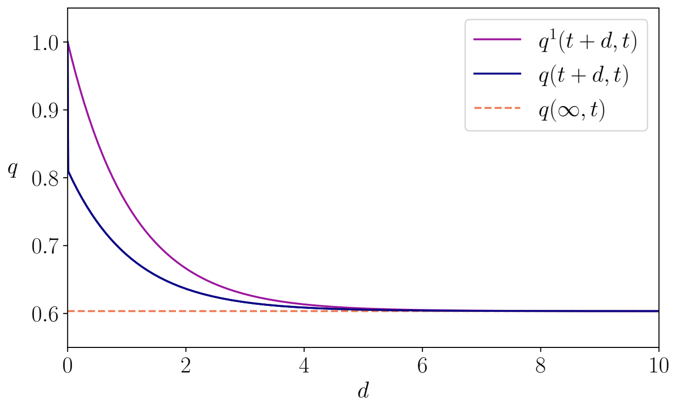

FIG. S1. Result of simulating Eq. S2.32,S2.33 to calculate $q(t+d,t)$ (blue line) and $q^1(t+d,t)$ (purple line) for $0 \leq d \leq 10$ with $\alpha = 0.01$, $\beta = 2$, $\Theta_{i,u} = 0$, $J_0 = 1$, and $\Delta J = 0.5$. We also calculated the long range limit using Eq. S2.40 (dashed orange line). We observe that both variables converge for large d to the result given by Eq. S2.40. In addition, we find that while $q^1(t+d,t)$ decays smoothly, $q(t+d,t)$ has a discontinuity at $d = 0$ since $q(t,t) = 1$.

Similarly, we define the continuous-time equivalent function

$$\begin{aligned} q_i(\infty,\infty) &\equiv \lim_{t\to\infty} q_i(\infty,t) = \lim_{t\to\infty} q_i^1(\infty,t) \\ &= \lim_{t\to\infty}\lim_{t'\to\infty} \int \mathrm{D}xy^{(q_i(t',t))} \tanh\left[\beta\left(\Theta_i + J_0 m(t') + \Delta J x\right)\right] \tanh\left[\beta\left(\Theta_i + J_0 m(t) + \Delta J y\right)\right]. \end{aligned} \tag{S2.40}$$

Note that Eqs. S2.27, S2.40 converge to the same values as Eqs. S1.69, S1.70 when the magnetic fields are equal to Θ_i, proving that the synchronous and asynchronous asymmetric SK models have identical solutions for their order parameters.

In Fig. S1, we numerically confirmed our theoretical result by simulating an exemplary dynamics of the self-correlation order parameters in the continuous-time domain. The procedure is as follows. We select an Euler step of $\alpha = 0.01$ and an initial values of $q_i(t',0) = \delta[d]$. Given $q_i^1(0,0) = 1$, we computed a forward pass of the values of $q_i^1(d,0)$ for larger values of d ($d \geq 0$), using Eq. S2.33. Then, given $t' = t+d$ we calculated one Euler step of Eq. S2.32 in t, updating the value of $q_i(t+d,t)$ for all values of d. We iterated the above procedure until the function $q_i(t+d,t)$ and $q_i^1(t+d,t)$ converge to fixed values. We confirmed that the convergence value of the process was the same one as directly calculating $q_i(\infty,\infty)$.

Entropy production

Assume that time-constant fields Θ_i, i.e., the fields $H_{i,u} = \Theta_i + (1-\tau_{i,u})Ks_{i,u-1}$ are stochastic processes driven by time-independent $\tau_{i,u}$. In this case the average difference of the forward and reverse entropy rates in Eqs. S1.71, S1.72 converges to:

$$\begin{aligned} [\sigma_u]_{\mathbf{J},\boldsymbol{\tau}} &= \sum_{\boldsymbol{\tau}_u,\boldsymbol{\tau}_{u-1}} p(\boldsymbol{\tau}_u)p(\boldsymbol{\tau}_{u-1})\left(S_{u|u-1} - S^r_{u|u-1}\right) \\ &= \sum_{\boldsymbol{\tau}_u} p(\boldsymbol{\tau}_u)\left(\beta^2\Delta J^2(1-q_{u,u-2})\sum_i \int \mathrm{D}z(1-\tanh^2\left[\beta\left(H_{i,u} + J_0 m_{u-1} + \Delta J z\right)\right])\right), \end{aligned} \tag{S2.41}$$

for the steady-state values of m_{u-1} and $q_{u,u-2}$ (independent of u).

For asynchronous updates in a steady state, non-zero contributions to the entropy production at spin i only occur during spin updates, i.e., $\tau_{i,u} = 1$ occurring with a probability α (we can see this in the equation above where the $\tanh^2\left[\beta\left(H_{i,u} + J_0 m + \Delta J z\right)\right]$ term becomes equal to 1 when $\tau_{i,u} = 0$). This leads to the steady-state entropy production value of

$$[\sigma_u]_{\mathbf{J},\boldsymbol{\tau}} = \alpha\beta^2\Delta J^2(1-q_{u,u-2})\sum_i \int \mathrm{D}z(1-\tanh^2\left[\beta\left(\Theta_i + J_0 m_u + \Delta J z\right)\right]). \tag{S2.42}$$

In the continuous-time limit $\alpha \to 0$, with a change of variables $t = (u-1)\alpha$, we have the entropy production rate defined in continuous time:

$$\left[\frac{d\sigma(t)}{dt}\right]_{\mathbf{J},\boldsymbol{\tau}} = \lim_{\alpha\to 0} \beta^2 \Delta J^2 (1 - q(t+\alpha, t-\alpha)) \sum_i \int \mathrm{D}z (1 - \tanh^2 \left[\beta \left(\Theta_i + J_0 m(t) + \Delta J z\right)\right]). \tag{S2.43}$$

Note that due to the discontinuity of $q(t+d, t)$ (or $q(t+d, t-d)$ equivalently), the limit in $d \to 0$ is different from $q(t,t) = 1$ (see Fig. S1). This property, $\lim_{d\to 0} q(t+d, t-d) < 1$, guarantees that the entropy production rate can be non-zero for the adequate parameters.

Supplementary Note 3: Ferromagnetic critical phase transition in the infinite kinetic Ising model with Gaussian couplings and uniform weights

We define a kinetic Ising network of infinite size under synchronous updates ($\alpha = 1$), with random fields $H_{i,u} = H_i$, where H_i are uniformly distributed following $\mathcal{U}(-\Delta H, \Delta H)$, and couplings J_{ij} follow a Gaussian distribution $\mathcal{N}(\frac{1}{N}, \frac{\Delta J^2}{N})$.

As we have found that the asymmetric SK model with arbitrary fields follows a mean-field solution, calculating the effects of disorder in the fields becomes easier, as we can approximate the update equations of the order parameters in the thermodynamic limit $N \to \infty$ as an integral with a large number of units:

$$\begin{aligned} m_u = \frac{1}{N}\sum_i m_{i,u} =& \frac{1}{2\Delta H}\int_{-\Delta H}^{\Delta H} dh \int \mathrm{D}z \tanh\left[\beta\left(h + J_0 m_{u-1} + \Delta J z\right)\right] \\ =& \frac{1}{2\beta\Delta H}\int \mathrm{D}z \log\frac{\cosh\left[\beta\left(\Delta H + J_0 m_{u-1} + \Delta J z\right)\right]}{\cosh\left[\beta\left(-\Delta H + J_0 m_{u-1} + \Delta J z\right)\right]}. \end{aligned} \tag{S3.1}$$

Similarly, the delayed self-correlation parameter:

$$\begin{aligned} q_{u,v} = \frac{1}{N}\sum_i R_{ii,u,v} =& \frac{1}{2\Delta H}\int_{-\Delta H}^{\Delta H} dh \int \mathrm{D}xy^{(q_{u-1,v-1})} \tanh\left[\beta\left(h + J_0 m_{u-1} + \Delta J x\right)\right] \tanh\left[\beta\left(h + J_0 m_{v-1} + \Delta J y\right)\right] \\ =& 1 + \frac{1}{2\beta\Delta H}\int \mathrm{D}xy^{(q_{u-1,v-1})} \frac{\left(\mathrm{e}^{2H_{v,y}\beta} + \mathrm{e}^{2\beta H_{u,x}}\right)}{\left(\mathrm{e}^{2\beta H_{v,y}} - \mathrm{e}^{2\beta H_{u,x}}\right)} \log\left[\frac{\mathrm{e}^{-2\beta\Delta H} + \mathrm{e}^{2\beta H_{u,x}}}{\mathrm{e}^{2\beta\Delta H} + \mathrm{e}^{2\beta H_{u,x}}}\frac{\mathrm{e}^{2\beta\Delta H} + \mathrm{e}^{2\beta H_{v,y}}}{\mathrm{e}^{-2\beta\Delta H} + \mathrm{e}^{2\beta H_{v,y}}}\right], \end{aligned} \tag{S3.2}$$

with $H_{u,x} = J_0 m_{u-1} + \Delta J x$ and $H_{v,y} = J_0 m_{v-1} + \Delta J y$.

In the zero-temperature limit, $\beta \to \infty$, these expressions have the following forms:

$$m_u = \frac{1}{2\beta\Delta H}\int \mathrm{D}z\left(\left|\Delta H + J_0 m_{u-1} + \Delta J z\right| - \left|-\Delta H + J_0 m_{u-1} + \Delta J z\right|\right), \tag{S3.3}$$

$$q_{u,v} = 1 + \frac{1}{2\beta\Delta H}\int \mathrm{D}xy^{(q_{u-1,v-1})} \mathrm{sign}\left[H_{v,y} - H_{u,x}\right]\beta\Bigg(\left|\Delta H + H_{u,x}\right| - \left|\Delta H - H_{u,x}\right| - \left|\Delta H + H_{v,y}\right| + \left|\Delta H - H_{v,y}\right|\Bigg). \tag{S3.4}$$

Finally, the normalized conditional entropy in the thermodynamic limit and normalized reversed conditional entropy are given as

$$\begin{aligned} \left[\frac{1}{N}S_{u|u-1}\right]_{\mathbf{J}} =& \frac{1}{2\Delta H}\int_{-\Delta H}^{\Delta H} dh \int \mathrm{D}z\Big(\beta^2\Delta J^2(1 - \tanh^2\left[\beta\left(h + J_0 m_{u-1} + \Delta J z\right)\right]) \\ &+ \beta h \tanh\left[\beta\left(h + J_0 m_{u-1} + \Delta J z\right)\right] - \log\left[2\cosh\left[\beta\left(h + J_0 m_{u-1} + \Delta J z\right)\right]\right]\Big) + \beta J_0 m_u m_{u-1} \\ =& \frac{1}{2\beta\Delta H}\int \mathrm{D}z\Big(\beta^2\Delta J^2 \tanh\left[\beta\left(\Delta H + J_0 m_{u-1} + \Delta J z\right)\right] - \tanh\left[\beta\left(-\Delta H + J_0 m_{u-1} + \Delta J z\right)\right]) \\ &+ \frac{1}{2\beta\Delta H}\left(\varphi\left(\beta\Delta H, \beta J_0 m_{u-1} + \beta\Delta J z\right) - \varphi\left(-\beta\Delta H, \beta J_0 m_{u-1} + \beta\Delta J z\right)\right)\Big) + \beta J_0 m_u m_{u-1}, \end{aligned} \tag{S3.5}$$

and

$$\begin{aligned} \frac{1}{N}\left[S^r_{u|u-1}\right]_{\mathbf{J}} =& \frac{1}{2\Delta H}\int_{-\Delta H}^{\Delta H} dh \int \mathrm{D}z\Big(q_{u,u-2}\beta^2\Delta J^2(1 - \tanh^2\left[\beta\left(h + J_0 m_{u-2} + \Delta J z\right)\right]) \\ &- \log\left[2\cosh\left[\beta\left(h + J_0 m_u + \Delta J z\right)\right]\right]\Big)\Bigg) + \beta J_0 m_{u-1} m_{u+1} \\ =& \frac{1}{2\beta\Delta H}\int \mathrm{D}z\Big(\beta^2\Delta J^2 q_{u,u-2}(\tanh\left[\beta\left(\Delta H + J_0 m_{u-2} + \Delta J z\right)\right] - \tanh\left[\beta\left(-\Delta H + J_0 m_{u-2} + \Delta J z\right)\right]) \\ &+ \frac{1}{2\beta\Delta H}\left(\varphi\left(\beta\Delta H, \beta J_0 m_u + \beta\Delta J z\right) - \varphi\left(-\beta\Delta H, \beta J_0 m_u + \beta\Delta J z\right)\right)\Big) + \beta J_0 m_{u-1} m_{u+1}, \end{aligned} \tag{S3.6}$$

where we define

$$\varphi(h,w) = h\log\left[1+\exp\left[2h+2w\right]\right] + \mathrm{Li}_2\left[-\exp\left[2h+2w\right]\right] + hw \tag{S3.7}$$

with $\mathrm{Li}_s[x]$ being the polylogarithm function.

S3.1. Critical points

Assuming a nonequilibrium steady state in which $m_u = m_{u-1} = m$, we obtain the critical point of the system by computing the non-zero solutions of the first order Taylor expansion around $m = 0$ of the right-hand part of Eq. S3.1,

$$\begin{aligned} m \approx& \frac{1}{2\beta\Delta H}\int \mathrm{D}z \log\frac{\cosh\left[\beta\left(\Delta H + \Delta J z\right)\right]}{\cosh\left[\beta\left(-\Delta H + \Delta J z\right)\right]} \\ &+ \frac{1}{2\beta\Delta H}\int \mathrm{D}z \left(\tanh\left[\beta\left(\Delta H + \Delta J z\right)\right] - \tanh\left[\beta\left(-\Delta H + \Delta J z\right)\right]\right)\beta J_0 m \\ =& \frac{1}{\Delta H}\int \mathrm{D}z \tanh\left[\beta\left(\Delta H + \Delta J z\right)\right] J_0 m. \end{aligned} \tag{S3.8}$$

This equation yields the self-consistent equation whose solution gives the critical inverse temperature, β_c:

$$\frac{\Delta H}{J_0} = \int \mathrm{D}z \tanh\left[\beta\left(\Delta H + \Delta J z\right)\right]. \tag{S3.9}$$

In the special case where $\Delta H = 0$, the expansion around $m = 0$ results in

$$\begin{aligned} m \approx& \int \mathrm{D}z \tanh\left[\beta\left(\Delta J z\right)\right] + \int \mathrm{D}z \left(1 - \tanh^2\left[\beta\left(\Delta J z\right)\right]\right)\beta J_0 m \\ =& \int \mathrm{D}z \left(1 - \tanh^2\left[\beta\left(\Delta J z\right)\right]\right)\beta J_0 m. \end{aligned} \tag{S3.10}$$

The critical value β_c is given by the solution of the equation,

$$\frac{1}{\beta J_0} = \int \mathrm{D}z \left(1 - \tanh^2\left[\beta\left(\Delta J z\right)\right]\right). \tag{S3.11}$$

Similarly, we can find the critical value of ΔJ at the limit of zero temperature by solving the equation in the $\beta \to \infty$ limit:

$$\frac{1}{\Delta H}\int \mathrm{D}z\, \mathrm{sign}\left[\left(\Delta H + \Delta J z\right)\right] J_0 = 1. \tag{S3.12}$$

S3.2. Critical exponents

We can characterize critical exponents of the system using the normalized inverse temperature $\tau = -\frac{\beta-\beta_c}{\beta_c}$. We first note that the first order Taylor expansion of the following term around the critical β_c yields

$$\begin{aligned} \frac{1}{\Delta H}\int \mathrm{D}z \tanh\left[\beta\left(\Delta H + \Delta J z\right)\right] J_0 \approx& 1 + \frac{1}{\Delta H}\int \mathrm{D}z \left(1 - \tanh^2\left(\beta_c\left(\Delta H + \Delta J z\right)\right)\right)\left(\Delta H + \Delta J z\right) J_0 \left(\beta - \beta_c\right) \\ =& 1 - K'\left(\beta - \beta_c\right). \end{aligned} \tag{S3.13}$$

We also note that the value of m around $\beta = \beta_c$ with the third order Taylor expansion is given as

$$\begin{aligned} m \approx& \frac{1}{\Delta H}\int \mathrm{D}z \tanh\left[\beta\left(\Delta H + \Delta J z\right)\right] J_0 m \\ &- \frac{1}{3\beta\Delta H}\int \mathrm{D}z \tanh\left[\beta\left(\Delta H + \Delta J z\right)\right]\left\{1 - \tanh^2\left[\beta\left(\Delta H + \Delta J z\right)\right]\right\}\left(\beta J_0 m\right)^3 \\ =& (1 - K'\left(\beta - \beta_c\right))m - K'' m^3, \end{aligned} \tag{S3.14}$$

from which we obtain

$$m \propto (\beta - \beta_c)^{\frac{1}{2}} \,. \tag{S3.15}$$

Thus we have a critical exponent $\frac{1}{2}$, which is consistent with the scaling exponent of the order parameter of the mean-field universality class typically denoted by the symbol 'β' in the literature.

Similarly, we can compute the susceptibility to a uniform magnetic field B added on top of H_i, having that

$$\begin{aligned} \frac{\partial m}{\partial B}\Big|_{B=0} =& \frac{1}{2\beta\Delta H} \int \mathrm{D}z \left\{ \tanh\left[\beta\left(\Delta H + \Delta J z\right)\right] - \tanh\left[\beta\left(-\Delta H + \Delta J z\right)\right] \right\} \left(\beta + \beta J_0 \frac{\partial m}{\partial B}\Big|_{B=0} \right) \\ =& \frac{1}{\Delta H} \int \mathrm{D}z \left\{ \tanh\left[\beta\left(\Delta H + \Delta J z\right)\right] \right\} \left(1 + J_0 \frac{\partial m}{\partial B}\Big|_{B=0} \right), \end{aligned} \tag{S3.16}$$

which evaluated at the limit $\tau \to 0$ results in

$$\frac{\partial m}{\partial B}\Big|_{B=0} = (1 - K'\tau) \left(\frac{1}{J_0} + \frac{\partial m}{\partial B} \right), \tag{S3.17}$$

$$\frac{\partial m}{\partial B}\Big|_{B=0} \propto \frac{1 - K'\tau}{\tau} \approx (-\tau)^{-1}, \tag{S3.18}$$

retrieving the $\gamma = 1$ exponent that is consistent with the mean-field universality class.

Note that these critical exponents, corresponding to the mean-field universality class, are also the same found in the order-disorder phase transition of the equilibrium SK model [12]. Note that the spin-glass phase, not present for asymmetric couplings, has different exponents and does not correspond to this universality class [52]).

Supplementary Note 4: Comparison with the equilibrium SK model

To illustrate distinct behaviors between the symmetric and asymmetric SK models, we compare the order parameters of the asymmetric SK model with those of its equilibrium counterparts. We use the replica-symmetric solution of the model [12], which becomes unstable for the spin-glass phase but still yields an approximate phase diagram of the system. Figure S2 displays the phase diagram of the order parameters of an equilibrium SK model, which is equivalent to that of the nonequilibrium SK model in the main text shown in Fig. 2.

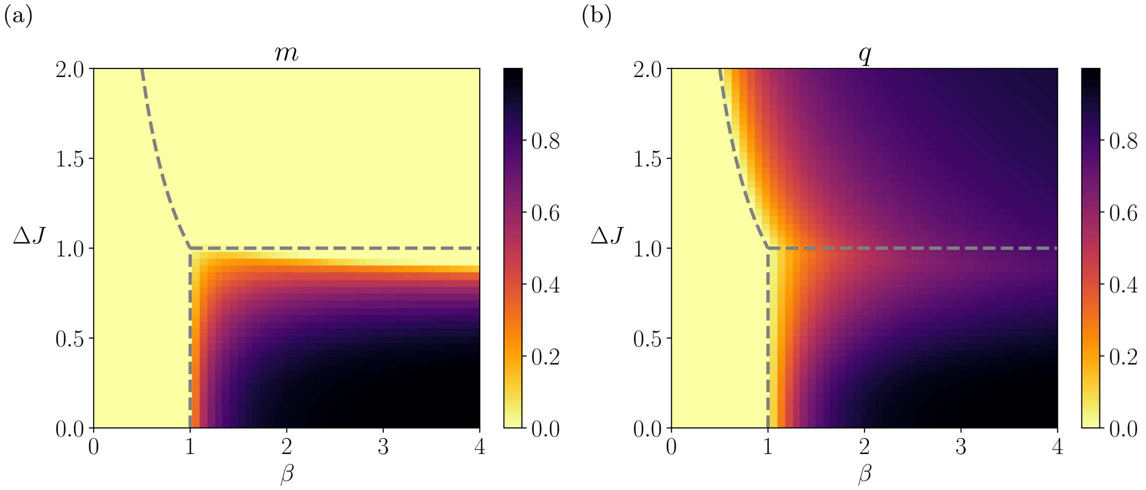

FIG. S2. **Order parameters of the equilibrium SK model with zero fields**. An approximate solution of the model with symmetric couplings is calculated under the replica-symmetry assumption [12]. The average magnetization m and the average delayed self-coupling q are shown for a model with fixed parameters $J_0 = 1$, $\Delta H = 0$ and variable ΔJ and β. The dashed line represents the critical line separating disordered (left), ordered (bottom-right) and spin-glass (top-right) phases.

Supplementary Note 5: Convergence times

Spin glasses show a particular slow decay functions, which converge non-exponentially following a non-trivial slow function [65]. This finding is replicated in models like the equilibrium SK model [54, 66]. To refute the existence of a spin-glass phase with such slow non-exponential decay, we simulated the convergence of the average magnetization as the dynamics reaches a nonequilibrium steady state. Using the critical inverse temperature β_c for $\Delta J = 0.2, \Delta H = 0$, we use 11 values of ΔJ uniformly distributed in the interval $[0.19, 0.21]$. In Fig. S3, we observe, at the critical value ($\Delta J = 0.2$, black line), the convergence of magnetization follows a power law, as expected. Conversely, both the ordered and disordered phases ($\Delta J < 0.2$, dotted line, $\Delta J > 0.2$, dashed line) converge as exponential functions. The figure is calculated for $\alpha = 1$, but the behavior is similar for all α. These results confirm that the disordered phase is not a spin-glass phase, as spin glasses show a non-exponential slow decay characterized by a non-trivial function.

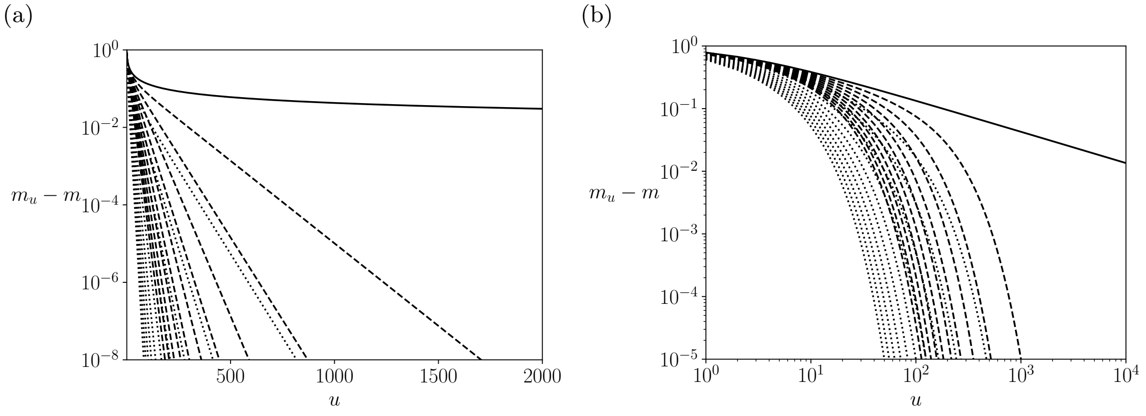

FIG. S3. **Convergence time**. Convergence times of the average-rate order parameter m_u to its stationary state value m at the critical point (black), ordered phase (dotted line) and disordered phase (dashed line) for a semilog (a) and log-log scales (b). All lines show an exponential decay, except for the system at criticality, which shows a power-law decay. We can know that the disordered phase is not a spin-glass phase due to the exponential decay.

Supplementary Note 6: Numerical simulations

To verify our theoretical solutions of the order parameters and entropy production obtained under the configurational average, we ran numerical simulations of the kinetic Ising systems with synchronous and asynchronous updates for many random realizations of the model parameters. Here we explain how we performed the numerical simulations and calculated the statistics from the sample trajectories.

First, we constructed a fully asymmetric kinetic Ising system of size N with random couplings. The elements J_{ij} of the coupling matrix $\mathbf{J}$ were randomly sampled from independent Gaussian distributions, $J_{ij} \sim \mathcal{N}\left(\frac{J_0}{N}, \frac{\Delta J}{N}\right)$. We simulated trajectories of $t = 2^7$ steps for synchronous updates and $T = 2^7 N$ steps for asynchronous updates (see in the following) for 101 values of the inverse temperature β in the range $[0, 4]$. For each system with size N, we run the simulations using $R = 400{,}000$ random configurations of the matrix $\mathbf{J}$, and we further repeated the process for different system sizes.

In the simulation, we updated each spin in accordance to the transition probability:

$$p(s_{i,u} \,|\, \mathbf{s}_{u-1}) = \frac{\exp\left[\beta s_{i,u} h_{i,u}\right]}{2\cosh\left[\beta h_{i,u}\right]}, \tag{S6.1}$$

$$h_{i,u} = \Theta_i + \sum_j J_{ij} s_{j,u-1}. \tag{S6.2}$$

For the Ising systems with synchronous updates, we simultaneously updated all spins at each time step, which is equivalent to setting $\alpha = 1$ in Eq. 34. For asynchronous updates, we randomly selected a single spin at each time step and updated the selected spin using the above equation to capture the behavior of the system in the continuous time limit with $\alpha \to 0$ and $K \to \infty$. In this time limit, updates will be infrequent and only one spin is updated at a time. That is, most of the time $\tau_{i,u} = 0$. However, for computational efficiency we only simulated the steps where a spin is updated with $\tau_{i,u} = 1$ (a random event happening with probability $N\alpha$). Thus, t steps of the system with synchronous updates in Eq. 34 with $\alpha \to 0$ corresponds to T steps in our simulation, where T is a stochastic variable corresponding to a Binomial distribution $B(Nt, \alpha)$ (which in the limit $\alpha \to 0$ is equivalent to a Poisson distribution $\mathrm{Pois}(Nt\alpha)$). To make the behavior of the synchronous and asynchronous system have equivalent speeds, we choose to use $t = 2^7$ for the synchronous system, and $T = 2^7 N$ for the asynchronous systems, approximately corresponding to $t = 2^7/\alpha$. This guarantees that we have a total of $2^7 N$ individual spin updates for both systems.

Next, we computed the order parameters and entropy production at a steady state from the sample trajectories as follows. We calculated the steady-state average activation rate from the last time step t of the sample trajectories as:

$$\widehat{m} = \frac{1}{N} \sum_i \left[\langle s_{i,t} \rangle\right]_{\mathbf{J},\boldsymbol{\tau}}, \tag{S6.3}$$

where $\langle \cdot \rangle_{\mathbf{J}}$ is an average over the R configurations of $\mathbf{J}$. The last time step t is $t = 2^7$ for the synchronous updates and $\frac{1}{\alpha} 2^7$ for asynchronous updates (corresponding to $2^7 N$ updates in our simulation) and large enough to make the systems reach the steady state.

We computed the steady-state delayed self-correlation from the samples in a similar way. For the synchronous Ising systems, it was computed as:

$$\widehat{q} = \frac{1}{N} \sum_i \langle s_{i,t} s_{i,t-1} \rangle_{\mathbf{J}}, \tag{S6.4}$$

with $\alpha = 1$ whereas in the asynchronous Ising model we used

$$\widehat{q} = \frac{1}{N} \sum_i \left[\langle s_{i,t} s_{i,t-d} \rangle\right]_{\mathbf{J},\boldsymbol{\tau}} \tag{S6.5}$$

with $d = 10/\alpha$ (with $t - d$ corresponding to the point $T - 10N$ in our simulation) to obtain the delayed correlation of the spin states between two distant time points (i.e., a long-range correlation).

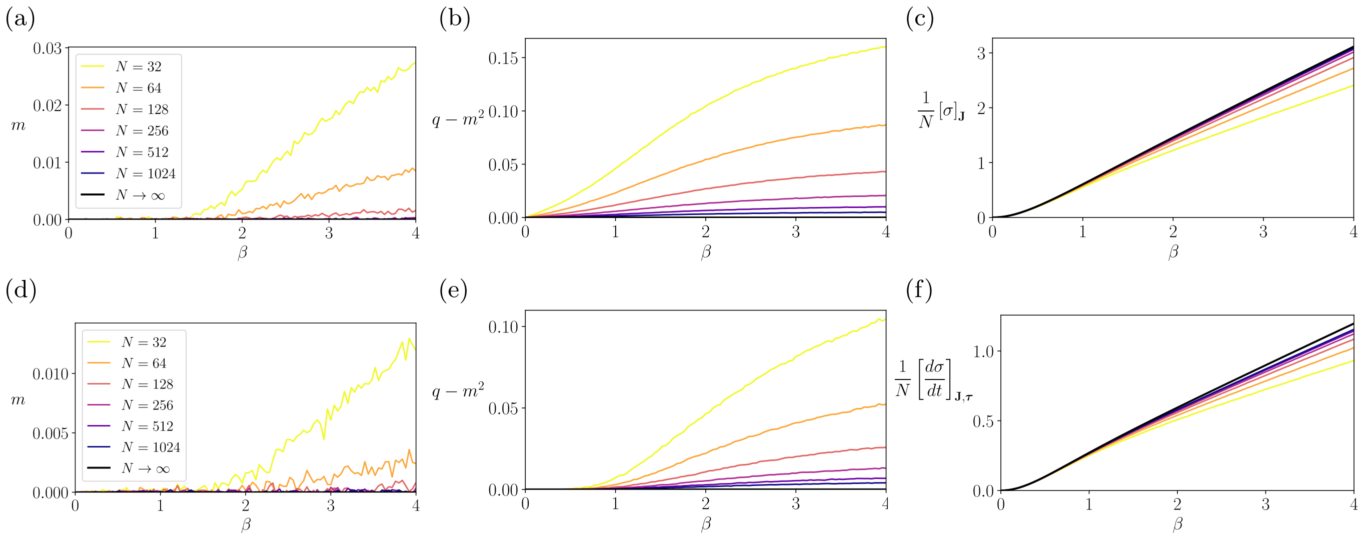

FIG. S4. **Verification of the exact mean-field solutions by simulating the kinetic Ising systems**. We repeated the simulations for systems of size $N = 32, 64, 128, 256, 512, 1024$ with synchronous (top) and asynchronous (bottom) updates with $\Theta_{i,u} = 0$ and $\Delta J = 1$. (a,d) Sampling estimation of the mean activation rate $\widehat{m}$ compared with the theoretical order parameter m (black lines). (b,e) Sampling estimation of the average delayed self-correlations $\widehat{q}$ compared with the theoretical order parameter q (black lines). (c,f) Sampling estimation of the entropy production and entropy production rate $\widehat{\sigma}, \lim_{\alpha\to 0}\frac{1}{\alpha}\widehat{\sigma}$ computed from the sample trajectories (Eq. S6.6, S6.7) compared with its mean-field value at the thermodynamic limit $\frac{1}{N}[\sigma]_{\mathbf{J}}, \frac{1}{N}\left[\frac{d\sigma}{dt}\right]_{\mathbf{J},\boldsymbol{\tau}}$ (black lines, Eqs. 54,58).

Finally, we calculated the steady-state entropy production from the samples using

$$\begin{aligned}\widehat{\sigma}_t =&\alpha\left[\left\langle\sum_i \log\frac{p(s_{i,t}\,|\,\mathbf{s}_{t-1})}{p(s_{i,t-1}\,|\,\mathbf{s}_t)}\right\rangle\right]_{\mathbf{J},\boldsymbol{\tau}}\\ =&\alpha\left[\left\langle\sum_i \Theta_i(s_{i,t}-s_{i,t-1}) + \sum_{ij} J_{ij}(s_{i,t}s_{j,t-1} - s_{i,t-1}s_{j,t})\right.\right.\\ &\left.\left.-\log(2\cosh(\Theta_i+\sum_j J_{ij}s_{j,t-1})) + \log(2\cosh(\Theta_i+\sum_j J_{ij}s_{j,t}))\right\rangle\right]_{\mathbf{J},\boldsymbol{\tau}}.\end{aligned}\tag{S6.6}$$

The steady-state entropy production of the synchronous update is obtained by setting $\alpha = 1$ and effectively removing the average over $\boldsymbol{\tau}$. Note in our results we normalize this entropy production by the number of spins N to make it independent of the system size.

The steady-state entropy rate for the asynchronous updates is given by $\lim_{\alpha\to 0}\widehat{\sigma}_t/\alpha$, to make it independent of the update rate α:

$$\begin{aligned}\lim_{\alpha\to 0}\frac{1}{\alpha}\widehat{\sigma}_t =&\left[\left\langle\sum_i \Theta_i(s_{i,t}-s_{i,t-1}) + \sum_{ij} J_{ij}(s_{i,t}s_{j,t-1} - s_{i,t-1}s_{j,t})\right.\right.\\ &\left.\left.-\log(2\cosh(\Theta_i+\sum_j J_{ij}s_{j,t-1})) + \log(2\cosh(\Theta_i+\sum_j J_{ij}s_{j,t}))\right\rangle\right]_{\mathbf{J},\boldsymbol{\tau}}.\end{aligned}\tag{S6.7}$$

Similarly to the synchronous case, we normalize this entropy production rate by the number of spins N to make it independent of the system size.

To verify our exact mean-field solutions, we simulated networks of different sizes with synchronous and asynchronous updates for the parameters $\Theta_{i,u} = 0$ and $\Delta J = 0.5$ (Fig. 4) or $\Delta J = 1$ (Fig. S4). These results corroborate our

theoretical predictions and confirm that the steady-state entropy production peaks at the phase transitions and increases when the significantly heterogeneous system approaches the quasi-deterministic regimes.

Supplementary Note 7: Dynamic patterns

To characterize the complexity of the spin kinetics, we count the number of states that returns to themselves after a number of transition steps with different lengths for a large but finite size system under steady state. We describe this probability as

$$\Omega^{(n)} = p(\mathbf{s}_u = \mathbf{s}_{u+n}, \mathbf{s}_u \neq \mathbf{s}_{u+n-1}, \mathbf{s}_u \neq \mathbf{s}_{u+n-2}, \dots). \tag{S7.1}$$

We will assume the synchronous Ising system with any n, or the asynchronous Ising system with large n, so the order parameters converge to their long-range values m, q.

Given this probability distribution, we can calculate the average transition length as

$$\sum_n n\Omega^{(n)}. \tag{S7.2}$$

A transition length of 1 results in a static pattern, where the same pattern appears consecutively. A transition length longer than 1 indicates that the dynamics exhibits a cyclic pattern on average. For simplicity, we assume that there are no fields $\Theta_{i,u} = 0$. Also, under steady state for the configurational average, the self-correlations are uniform across elements, having $[\langle s_{i,u} s_{i,v}\rangle] = q$.

S7.1. The proportion of patterns with length n

The proportion for the patterns with an arbitrary length n is obtained as follows. First, we note that by using a Kronecker delta $\delta[\mathbf{s}_u, \mathbf{s}_{u-k}]$, it can be written as

$$\begin{aligned}\Omega^{(n)} =& \sum_{\mathbf{s}_{0:t}} p(s_{0:t}) \delta[\mathbf{s}_u, \mathbf{s}_{u-n}] \prod_{k=1}^{n-1} (1 - \delta[\mathbf{s}_u, \mathbf{s}_{u-k}]) \\ =& \Psi^{(n-1)} - \Psi^{(n)},\end{aligned} \tag{S7.3}$$

$$\Psi^{(n)} = \sum_{\mathbf{s}_{0:t}} p(s_{0:t}) \prod_{k=1}^{n} (1 - \delta[\mathbf{s}_u, \mathbf{s}_{u-k}]). \tag{S7.4}$$

Here, $\Omega^{(n)}$ is the probability of observing $n-1$ patterns different from $\mathbf{s}_u$ during consecutive state updates and finally observing $\mathbf{s}_u$ at the n-th step. In turn, $\Psi^{(n)}$ is the probability of just observing n patterns different from $\mathbf{s}_u$ during n state updates. Both $\Omega^{(n)}$ and $\Psi^{(n)}$ are probability distributions that meet $\sum_{n=1}^{\infty} \Omega^{(n)} = 1$ and $\sum_{n=1}^{\infty} \Psi^{(n)} = 1$. This is guaranteed by defining $\Omega^{(1)} = 1 - \Psi^{(1)}$, so that $\sum_{n=1}^{\infty} \Omega^{(n)} = 1 - \Psi^{(\infty)} = 1$ (as the probability $\Psi^{(\infty)}$ converges to zero).

We can expand $\Psi^{(n)}$ as:

$$\begin{aligned}\Psi^{(n)} =& 1 - \sum_k \Delta_k + \sum_{k<l} \Delta_{k,l} - \sum_{k<l<m} \Delta_{k,l,m} + \dots, \\ \Delta_{k,l,m,\dots} =& \sum_{\mathbf{s}_{0:t}} p(s_{0:t}) \delta[\mathbf{s}_u, \mathbf{s}_{u-k}] \delta[\mathbf{s}_u, \mathbf{s}_{u-l}] \delta[\mathbf{s}_u, \mathbf{s}_{u-m}] \cdots .\end{aligned} \tag{S7.5}$$

In steady state, the product of $\delta[\mathbf{s}_u, \mathbf{s}_{u-k}] \delta[\mathbf{s}_u, \mathbf{s}_{u-l}] \delta[\mathbf{s}_u, \mathbf{s}_{u-m}] \cdots$ for any set of $k \neq l \neq m \cdots$ results in the same value. This simplifies the previous equation to

$$\Psi^{(n)} = \sum_{k=0}^{n} \binom{n}{k} (-1)^k \Delta^{(k)}, \tag{S7.6}$$

$$\Delta^{(k)} = \prod_{k'=1}^{k} \delta[\mathbf{s}_u, \mathbf{s}_{u-k'}]. \tag{S7.7}$$

Using $\delta[\mathbf{s}_u, \mathbf{s}_{u-k}] = \prod_i \frac{1+s_{i,u}s_{i,u-k}}{2}$, the configurational average of $\Delta^{(n)}$ becomes

$$\begin{aligned}\left[\Delta^{(n)}\right]_{\mathbf{J},\boldsymbol{\tau}} =& \left[\sum_{\mathbf{s}_{0:t}} p(s_{0:t}) \prod_{k=1}^{n}\prod_{i=1}^{N} \frac{1+s_{i,u}s_{i,u-k}}{2}\right]_{\mathbf{J},\boldsymbol{\tau}} \\ =& \frac{1}{2^{nN}}\left[\left\langle \prod_{i=1}^{N}\prod_{k=1}^{n}(1+s_{i,u}s_{i,u-k})\right\rangle\right]_{\mathbf{J},\boldsymbol{\tau}} \\ =& \frac{1}{2^{nN}}\left[\left\langle \prod_{i=1}^{N}(1+s_{i,u}s_{i,u-1})(1+s_{i,u}s_{i,u-2})\cdots(1+s_{i,u}s_{i,u-n})\right\rangle\right]_{\mathbf{J},\boldsymbol{\tau}} \\ =& \frac{1}{2^{nN}}\left[\left\langle \prod_{i=1}^{N}\sum_{k=0}^{n}\binom{n}{k}(s_{i,u})^k s_{i,u-i_1}s_{i,u-i_2}\cdots s_{i,u-i_k}\right\rangle\right]_{\mathbf{J},\boldsymbol{\tau}},\end{aligned} \tag{S7.8}$$

where $\{i_1, i_2, \ldots, i_k\}$ are the set of k indices chosen form $1, \ldots, n$. Since $s_{i,u}s_{i,u} = 1$, $(s_{i,u})^k$ is 1 when k is even and $s_{i,u}$ when k is an odd number. It can be summarized as

$$(s_{i,u})^k = \frac{1+(-1)^k}{2} + s_{i,u}\frac{1-(-1)^k}{2}. \tag{S7.9}$$

Using this equation, we obtain

$$\begin{aligned}\left[\Delta^{(n)}\right]_{\mathbf{J},\boldsymbol{\tau}} =& \frac{1}{2^{nN}}\left[\left\langle \prod_{i=1}^{N}\sum_{k=0}^{n}\binom{n}{k}\left(\frac{1+(-1)^k}{2} + s_{i,u}\frac{1-(-1)^k}{2}\right)\prod_{l=1}^{k}s_{i,u-i_l}\right\rangle\right]_{\mathbf{J},\boldsymbol{\tau}} \\ =& \frac{1}{2^{nN}}\prod_{i=1}^{N}\sum_{k=0}^{n}\binom{n}{k}\left(\frac{1+(-1)^k}{2}\left[\left\langle\prod_{l=1}^{k}s_{i,u-i_l}\right\rangle\right]_{\mathbf{J},\boldsymbol{\tau}} + \frac{1-(-1)^k}{2}\left[\left\langle s_{i,u}\prod_{l=1}^{k}s_{i,u-i_l}\right\rangle\right]_{\mathbf{J},\boldsymbol{\tau}}\right).\end{aligned} \tag{S7.10}$$

The expression above is difficult to compute in general without resorting to extra assumptions, but we can illustrate its behavior for small pattern lengths.

The proportion of static patterns (1-periodic) in the system in steady state (in large t limit) can be calculated as

$$\begin{aligned}\Omega^{(1)} =& 1 - \Psi^{(1)} = \sum_{\mathbf{s}_{0:t}} p(s_{0:t})\delta[\mathbf{s}_u, \mathbf{s}_{u-1}] \\ =& \sum_{\mathbf{s}_{0:t}} p(s_{0:t})\prod_i \frac{1+s_{i,u}s_{i,u-1}}{2}.\end{aligned} \tag{S7.11}$$

The configurational average of the proportion is

$$\left[\Omega^{(1)}\right]_{\mathbf{J},\boldsymbol{\tau}} = \left(\frac{1+q}{2}\right)^N. \tag{S7.12}$$

That is, for $q = 1$ (e.g., $\Delta J = 0$ and $\beta \to \infty$), the system will display only static patterns.

The proportion of 2-periodic patterns can be calculated as

$$\begin{aligned}\Omega^{(2)} =& \Psi^{(1)} - \Psi^{(2)} = \sum_{\mathbf{s}_{0:t}} p(s_{0:t})\delta[\mathbf{s}_u, \mathbf{s}_{u-2}](1-\delta[\mathbf{s}_u, \mathbf{s}_{u-1}]) \\ =& \sum_{\mathbf{s}_{0:t}} p(s_{0:t})\prod_i \frac{1+s_{i,u}s_{i,u-2}}{2}\left(1 - \prod_i \frac{1+s_{i,u}s_{i,u-1}}{2}\right),\end{aligned} \tag{S7.13}$$

$$\left[\Omega^{(2)}\right]_{\mathbf{J},\boldsymbol{\tau}} = 2^{-N}\left((1+q)^N - \left(\frac{1+3q}{2}\right)^N\right) = \left[\Omega^{(1)}\right]_{\mathbf{J},\boldsymbol{\tau}}\left(1 - \left(\frac{1+3q}{2+2q}\right)^N\right). \tag{S7.14}$$

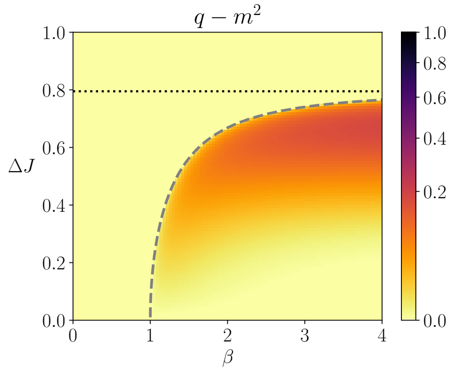

FIG. S5. Correlated activity in the configurational average, captured by spin covariances $q-m^2$. We observe that spins become independent both in the disordered phase and in the *deep* ordered phase.

Similarly, the proportion of 3-periodic patterns is

$$\Omega^{(3)} = \Psi^{(2)} - \Psi^{(3)} = \sum_{\mathbf{s}_{0:t}} p(s_{0:t})\delta[\mathbf{s}_u, \mathbf{s}_{u-3}](1-\delta[\mathbf{s}_u, \mathbf{s}_{u-1}])(1-\delta[\mathbf{s}_u, \mathbf{s}_{u-2}]))$$

$$= \sum_{\mathbf{s}_{0:t}} p(s_{0:t}) \prod_i \frac{1+s_{i,u}s_{i,u-3}}{2}\left(1-\prod_i \frac{1+s_{i,u}s_{i,u-1}}{2}\right)\left(1-\prod_i \frac{1+s_{i,u}s_{i,u-2}}{2}\right), \tag{S7.15}$$

$$\left[\Omega^{(3)}\right]_{\mathbf{J},\boldsymbol{\tau}} = 2^{-N}\left((1+q)^N - 2\left(\frac{1+3q}{2}\right)^N + \left(\frac{1+6q+\rho^{(3)}}{4}\right)^N\right)$$

$$= \left[\Omega^{(1)}\right]_{\mathbf{J},\boldsymbol{\tau}}\left(1-2\left(\frac{1+3q}{2+2q}\right)^N + \left(\frac{1+6q+\rho^{(3)}}{4+4q}\right)^N\right), \tag{S7.16}$$

where $\rho_t^{(3)} = [\langle s_{i,u}s_{i,u-1}s_{i,u-2}\rangle]$, which can be calculated as a three-dimensional Gaussian integral

$$\rho_u^{(3)} = \frac{1}{N}\sum_i \int p(\xi) \prod_{\tau=0}^{3} \tanh\left[\beta \overline{h}_{i,u-\tau}(\xi_{u-\tau})\right]. \tag{S7.17}$$

S7.2. Expected cycle length in the disordered and deep-ordered phases

As shown in Fig. S5, the system in steady state exhibits uncorrelated dynamics across the time steps ($q-m^2=0$) at the disordered phase ($\Delta J(\beta) > \Delta J^c(\beta)$) and at the limit of $\beta \to \infty$ in the ordered phase ($\Delta J(\beta) < \Delta J^c(\beta)$). We call the latter the deep ordered phase. For the uncorrelated dynamics, the configurational averages of spins in Eq. S7.10 are given by the product of m, which results in

$$\left[\Delta^{(n)}\right]_{\mathbf{J},\boldsymbol{\tau}} = \frac{1}{2^{nN}}\left(\sum_{k=0}^{n}\binom{n}{k}\left(\frac{1+(-1)^k}{2}m^k + \frac{1-(-1)^k}{2}m^{k+1}\right)\right)^N$$

$$= \frac{1}{2^{nN}}\left(\frac{1}{2}((1+m)^n + (1-m)^n + m(1+m)^n - m(1-m)^n)\right)^N$$

$$= \frac{1}{2^{(n+1)N}}\left((1+m)^{n+1} + (1-m)^{n+1}\right)^N. \tag{S7.18}$$

In the following, we provide the configurational average of the proportion for the disordered and deep-ordered phases.

a. Disordered phase

In the disordered phase, the mean activation rate is $m = 0$. This results in $\left[\Delta^{(n)}\right] = 2^{-nN}$. Hence we obtain

$$\left[\Psi^{(n)}\right]_{\mathbf{J},\boldsymbol{\tau}} = \sum_{k=0}^{n} \binom{n}{k} (-1)^k 2^{-kN} = \left(1 - 2^{-N}\right)^n, \tag{S7.19}$$

by using the binomial expansion formula. The configurational average of the proportion is computed as

$$\left[\Omega^{(n)}\right]_{\mathbf{J},\boldsymbol{\tau}} = 2^{-N} \left(1 - 2^{-N}\right)^{n-1} \approx \lambda \exp\left[(1-n)\lambda\right], \tag{S7.20}$$

where $\lambda = 2^{-N}$. The proportion of the longer cycle length exponentially decays with the rate 2^{-N}, and the average cycle length is given by $1/\lambda = 2^N$. Namely,

$$\sum_n n \left[\Omega^{(n)}\right]_{\mathbf{J},\boldsymbol{\tau}} = 2^N, \tag{S7.21}$$

which is the number of possible patterns.

b. Deep ordered phase

In the deep ordered phase, m is positive. We expect $(1+m)^{n+1} \gg (1-m)^{n+1}$ for sufficiently large m ($m \sim 1$). Under this condition, Eq. S7.18 is approximated as

$$\left[\Delta^{(n)}\right]_{\mathbf{J},\boldsymbol{\tau}} = \left(\frac{1+m}{2}\right)^{(n+1)N} \left(1 + \left(\frac{1-m}{1+m}\right)^{n+1}\right)^N \approx \left(\frac{1+m}{2}\right)^{(n+1)N}. \tag{S7.22}$$

Therefore, we obtain

$$\left[\Psi^{(n)}\right]_{\mathbf{J},\boldsymbol{\tau}} \approx Z^{-1} \left(\frac{1+m}{2}\right)^N \sum_{k=0}^{n} \binom{n}{k} (-1)^k \left(\frac{1+m}{2}\right)^{kN} = \left(1 - \left(\frac{1+m}{2}\right)^N\right)^n, \tag{S7.23}$$

by the binomial expansion formula. Here, we have introduced $Z = \sum_{k=0}^{n} \binom{n}{k} \left[\Delta^{(k)}\right]$ as a normalization factor to compensate for approximation errors and ensure the consistency condition $\sum_n \left[\Psi^{(n)}\right] = 1$ (which also guarantees by definition $\sum_n \left[\Omega^{(n)}\right] = 1$). Using this expression, we can compute the probabilities $\left[\Omega^{(n)}\right]$ of pattern lengths as

$$\left[\Omega^{(n)}\right] \approx \left(\left(\frac{1+m}{2}\right)^N \left(1 - \left(\frac{1+m}{2}\right)^N\right) \right)^{n-1} \approx \lambda \exp\left[(1-n)\lambda\right], \tag{S7.24}$$

where

$$\lambda = \left(\frac{1+m}{2}\right)^N. \tag{S7.25}$$

The last step is the exponential approximation of the geometric distribution guaranteed for a large N. In this case, the average pattern length is

$$\sum_n n \left[\Omega^{(n)}\right] \approx \frac{1}{\lambda} = \left(\frac{2}{1+m}\right)^N. \tag{S7.26}$$

The result reveals that the average pattern length grows exponentially with the system size N, and the growth rate becomes slower as m increases.

We note that the result for the deep ordered phase includes the result of disordered phase: inserting $m = 0$ yields $\lambda = 2^{-N}$ and the average pattern length $1/\lambda = 2^N$, which we found at the disordered phase.

---

[1] Wiener, N. Newtonian and Bergsonian time. In *Cybernetics, or control and communication in the animal and the machine, 2nd ed*, 30–44 (John Wiley & Sons Inc, Hoboken, NJ, US, 1961).

[2] Kondepudi, D. & Prigogine, I. *Modern thermodynamics: from heat engines to dissipative structures* (John Wiley & Sons, 2014).

[3] England, J. L. Statistical physics of self-replication. *The Journal of chemical physics* **139**, 09B623_1 (2013).

[4] Lynn, C. W., Cornblath, E. J., Papadopoulos, L., Bertolero, M. A. & Bassett, D. S. Broken detailed balance and entropy production in the human brain. *Proceedings of the National Academy of Sciences* **118** (2021).

[5] Sanz Perl, Y. *et al.* Nonequilibrium brain dynamics as a signature of consciousness. *Physical Review E* **104**, 014411 (2021).

[6] de la Fuente, L. A. *et al.* Temporal irreversibility of neural dynamics as a signature of consciousness. *Cerebral Cortex* (2022).

[7] Deco, G., Perl, Y. S., Sitt, J. D., Tagliazucchi, E. & Kringelbach, M. L. Deep learning the arrow of time in brain activity: Characterising brain-environment behavioural interactions in health and disease. *bioRxiv* (2021).

[8] Seifert, U. Stochastic thermodynamics, fluctuation theorems and molecular machines. *Reports on progress in physics* **75**, 126001 (2012).

[9] Herpich, T., Thingna, J. & Esposito, M. Collective power: minimal model for thermodynamics of nonequilibrium phase transitions. *Physical Review X* **8**, 031056 (2018).

[10] Suñé, M. & Imparato, A. Out-of-equilibrium clock model at the verge of criticality. *Physical Review Letters* **123**, 070601 (2019).

[11] Herpich, T., Cossetto, T., Falasco, G. & Esposito, M. Stochastic thermodynamics of all-to-all interacting many-body systems. *New Journal of Physics* **22**, 063005 (2020).

[12] Nishimori, H. *Statistical physics of spin glasses and information processing: an introduction* (Clarendon Press, 2001).

[13] Roudi, Y., Dunn, B. & Hertz, J. Multi-neuronal activity and functional connectivity in cell assemblies. *Current opinion in neurobiology* **32**, 38–44 (2015).

[14] Lang, A. H., Li, H., Collins, J. J. & Mehta, P. Epigenetic landscapes explain partially reprogrammed cells and identify key reprogramming genes. *PLoS computational biology* **10**, e1003734 (2014).

[15] Sherrington, D. & Kirkpatrick, S. Solvable model of a spin-glass. *Physical review letters* **35**, 1792 (1975).

[16] Parisi, G. A sequence of approximated solutions to the sk model for spin glasses. *Journal of Physics A: Mathematical and General* **13**, L115 (1980).

[17] Brunetti, R., Parisi, G. & Ritort, F. Asymmetric Little spin-glass model. *Physical Review B* **46**, 5339–5350 (1992).

[18] Schnakenberg, J. Network theory of microscopic and macroscopic behavior of master equation systems. *Reviews of Modern Physics* **48**, 571–585 (1976).

[19] Gaspard, P. Time-reversed dynamical entropy and irreversibility in markovian random processes. *Journal of statistical physics* **117**, 599–615 (2004).

[20] Ito, S., Oizumi, M. & Amari, S.-i. Unified framework for the entropy production and the stochastic interaction based on information geometry. *Physical Review Research* **2**, 033048 (2020).

[21] Jarzynski, C. Hamiltonian Derivation of a Detailed Fluctuation Theorem. *Journal of Statistical Physics* **98**, 77–102 (2000).

[22] Maes, C. Local detailed balance. *SciPost Physics Lecture Notes* 032 (2021).

[23] Crisanti, A. & Sompolinsky, H. Path integral approach to random neural networks. *Physical Review E* **98**, 062120 (2018).

[24] Crisanti, A. & Sompolinsky, H. Dynamics of spin systems with randomly asymmetric bonds: Langevin dynamics and a spherical model. *Physical Review A* **36**, 4922 (1987).

[25] Crisanti, A. & Sompolinsky, H. Dynamics of spin systems with randomly asymmetric bonds: Ising spins and glauber dynamics. *Physical Review A* **37**, 4865 (1988).

[26] Falasco, G., Rao, R. & Esposito, M. Information thermodynamics of turing patterns. *Physical review letters* **121**, 108301 (2018).

[27] Nguyen, B., Seifert, U. & Barato, A. C. Phase transition in thermodynamically consistent biochemical oscillators. *The Journal of chemical physics* **149**, 045101 (2018).

[28] Zhang, Y. & Barato, A. C. Critical behavior of entropy production and learning rate: Ising model with an oscillating field. *Journal of Statistical Mechanics: Theory and Experiment* **2016**, 113207 (2016).

[29] Noa, C. E. F., Harunari, P. E., de Oliveira, M. J. & Fiore, C. E. Entropy production as a tool for characterizing nonequilibrium phase transitions. *Physical Review E* **100**, 012104 (2019).

[30] Crochik, L. & Tomé, T. Entropy production in the majority-vote model. *Physical Review E* **72**, 057103 (2005).

[31] Aguilera, M., Moosavi, S. A. & Shimazaki, H. A unifying framework for mean-field theories of asymmetric kinetic ising systems. *Nature communications* **12**, 1–12 (2021).

[32] Jaynes, E. T. *Probability theory: The logic of science* (Cambridge university press, 2003).

[33] Jaynes, E. T. Macroscopic prediction. In *Complex Systems—Operational Approaches in Neurobiology, Physics, and Computers*, 254–269 (Springer, 1985).

[34] Pressé, S., Ghosh, K., Lee, J. & Dill, K. A. Principles of maximum entropy and maximum caliber in statistical physics. *Reviews of Modern Physics* **85**, 1115 (2013).

[35] Ge, H., Pressé, S., Ghosh, K. & Dill, K. A. Markov processes follow from the principle of maximum caliber. *The Journal of chemical physics* **136**, 064108 (2012).

[36] Livesey, A. K. & Skilling, J. Maximum entropy theory. *Acta Crystallographica Section A: Foundations of Crystallography*

41, 113–122 (1985).
[37] Kolomogoroff, A. *Grundbegriffe der wahrscheinlichkeitsrechnung*, vol. 2 (Springer-Verlag, 2013).
[38] Freedman, A. Convergence theorem for finite markov chains. *Proc. REU* (2017).
[39] Van den Broeck, C. & Esposito, M. Ensemble and trajectory thermodynamics: A brief introduction. *Physica A: Statistical Mechanics and its Applications* **418**, 6–16 (2015).
[40] Crooks, G. E. Nonequilibrium measurements of free energy differences for microscopically reversible markovian systems. *Journal of Statistical Physics* **90**, 1481–1487 (1998).
[41] Esposito, M. & Van den Broeck, C. Three detailed fluctuation theorems. *Physical review letters* **104**, 090601 (2010).
[42] Igarashi, M. Entropy production for discrete-time markov processes. *arXiv* 2205.07214 (2022).
[43] Evans, D. J. & Searles, D. J. The fluctuation theorem. *Advances in Physics* **51**, 1529 – 1585 (2002).
[44] Yang, Y.-J. & Qian, H. Unified formalism for entropy production and fluctuation relations. *Physical Review E* **101**, 022129 (2020).
[45] Hatano, T. & Sasa, S.-i. Steady-state thermodynamics of langevin systems. *Physical review letters* **86**, 3463 (2001).
[46] Dechant, A., Sasa, S.-i. & Ito, S. Geometric decomposition of entropy production in out-of-equilibrium systems. *Physical Review Research* **4**, L012034 (2022).
[47] Touchette, H. The large deviation approach to statistical mechanics. *Physics Reports* **478**, 1–69 (2009).
[48] Touchette, H. & Harris, R. J. *Large Deviation Approach to Nonequilibrium Systems*, chap. 11, 335–360 (John Wiley & Sons, Ltd, 2013).
[49] Touchette, H. Introduction to dynamical large deviations of markov processes. *Physica A: Statistical Mechanics and its Applications* **504**, 5–19 (2018).
[50] Lecomte, V., Appert-Rolland, C. & van Wijland, F. Thermodynamic Formalism for Systems with Markov Dynamics. *Journal of Statistical Physics* **127**, 51–106 (2007).
[51] Hertz, J. A., Roudi, Y. & Sollich, P. Path integral methods for the dynamics of stochastic and disordered systems. *Journal of Physics A: Mathematical and Theoretical* **50**, 033001 (2016).
[52] Oppermann, R. & Schmidt, M. J. Universality class of replica symmetry breaking, scaling behavior, and the low-temperature fixed-point order function of the sherrington-kirkpatrick model. *Physical Review E* **78**, 061124 (2008).
[53] Parisi, G. Toward a mean field theory for spin glasses. *Physics Letters A* **73**, 203–205 (1979).
[54] Sompolinsky, H. & Zippelius, A. Dynamic theory of the spin-glass phase. *Physical Review Letters* **47**, 359 (1981).
[55] Sompolinsky, H. & Zippelius, A. Relaxational dynamics of the edwards-anderson model and the mean-field theory of spin-glasses. *Physical Review B* **25**, 6860 (1982).
[56] Eissfeller, H. & Opper, M. Mean-field monte carlo approach to the sherrington-kirkpatrick model with asymmetric couplings. *Physical Review E* **50**, 709 (1994).
[57] Eldar, E., Cohen, J. D. & Niv, Y. The effects of neural gain on attention and learning. *Nature neuroscience* **16**, 1146–1153 (2013).
[58] Abeles, M. *Corticonics: Neural circuits of the cerebral cortex* (Cambridge University Press, 1991).
[59] Diesmann, M., Gewaltig, M.-O. & Aertsen, A. Stable propagation of synchronous spiking in cortical neural networks. *Nature* **402**, 529–533 (1999).
[60] Ikegaya, Y. *et al.* Synfire chains and cortical songs: temporal modules of cortical activity. *Science* **304**, 559–564 (2004).
[61] Izhikevich, E. M. Polychronization: computation with spikes. *Neural computation* **18**, 245–282 (2006).
[62] Beggs, J. M. & Plenz, D. Neuronal avalanches in neocortical circuits. *Journal of neuroscience* **23**, 11167–11177 (2003).
[63] Friedman, N. *et al.* Universal critical dynamics in high resolution neuronal avalanche data. *Physical review letters* **108**, 208102 (2012).
[64] Donner, C., Obermayer, K. & Shimazaki, H. Approximate inference for time-varying interactions and macroscopic dynamics of neural populations. *PLoS computational biology* **13**, e1005309 (2017).
[65] Joy, P., Kumar, P. A. & Date, S. The relationship between field-cooled and zero-field-cooled susceptibilities of some ordered magnetic systems. *Journal of physics: condensed matter* **10**, 11049 (1998).
[66] Cugliandolo, L. F., Kurchan, J. & Ritort, F. Evidence of aging in spin-glass mean-field models. *Physical Review B* **49**, 6331 (1994).
